# Integrated epigenetic and genetic programming of primary human T cells

**DOI:** 10.1038/s41587-025-02856-w

**Published:** 2025-10-21

**Authors:** Laine Goudy, Alvin Ha, Ashir A. Borah, Jennifer M. Umhoefer, Lauren Chow, Carinna Tran, Aidan Winters, Alexis Talbot, Rosmely Hernandez, Zhongmei Li, Sanjana Subramanya, Abolfazl Arab, Nupura Kale, Jae Hyun J. Lee, Joseph J. Muldoon, Chang Liu, Ralf Schmidt, Philip Santangelo, Julia Carnevale, Justin Eyquem, Brian R. Shy, Alex Marson, Luke A. Gilbert

**Affiliations:** 1 Gladstone-UCSF Institute of Genomic Immunology, San Francisco, CA, USA.; 2 Arc Institute, Palo Alto, CA, USA.; 3 Department of Laboratory Medicine, University of California, San Francisco, San Francisco, CA, USA.; 4 Department of Medicine, University of California, San Francisco, San Francisco, CA, USA.; 5 Université de Paris Cité, INSERM UMR976, Hôpital St-Louis, Paris, France.; 6 Department of Laboratory Medicine, Medical University of Vienna, Vienna, Austria.; 7 Petit Institute for Bioengineering and Biosciences, Georgia Institute of Technology, Atlanta, GA, USA.; 8 Department of Chemical Engineering, Georgia Institute of Technology, Atlanta, GA, USA.; 9 Wallace H. Coulter Department of Biomedical Engineering, Georgia Institute of Technology, Atlanta, GA, USA.; 10 Parker Institute for Cancer Immunotherapy, San Francisco, CA, USA.; 11 UCSF Helen Diller Family Comprehensive Cancer Center, University of California, San Francisco, San Francisco, CA, USA.; 12 Department of Microbiology and Immunology, University of California, San Francisco, San Francisco, CA, USA.; 13 Innovative Genomics Institute, University of California, Berkeley, Berkeley, CA, USA.; 14 Department of Urology, University of California, San Francisco, San Francisco, CA, USA.

## Abstract

Targeted epigenetic engineering of gene expression in cell therapies would allow programming of desirable phenotypes without many of the challenges and safety risks associated with double-strand break-based genetic editing approaches. Here, we develop an all-RNA platform for efficient, durable and multiplexed epigenetic programming in primary human T cells, stably turning endogenous genes off or on using CRISPRoff and CRISPRon epigenetic editors. We achieve epigenetic programming of diverse targeted genomic elements without the need for sustained expression of CRISPR systems. CRISPRoff-mediated gene silencing is maintained through numerous cell divisions, T cell stimulations and in vivo adoptive transfer, avoiding cytotoxicity or chromosomal abnormalities inherent to multiplexed Cas9-mediated genome editing. Lastly, we successfully combined genetic and epigenetic engineering using orthogonal CRISPR Cas12a–dCas9 systems for targeted chimeric antigen receptor (CAR) knock-in and CRISPRoff silencing of therapeutically relevant genes to improve preclinical CAR-T cell-mediated in vivo tumor control and survival.

Engineered T cells, containing transgenic T cell receptors (TCRs), chimeric antigen receptors (CARs) or other synthetic antigen receptors, are an emerging modality to treat cancer, autoimmunity and infectious diseases^1–4^. Although autologous CAR-T cells have been transformative for treating aggressive hematological malignancies, substantial advances are needed to achieve similar success in treating solid tumors and generating allogeneic cell therapies. Solid tumors demonstrate a number of challenges including immunosuppressive tumor microenvironments, physical barriers and T cell exhaustion that limit the responses of current therapies^5–7^. Allogeneic CAR-T cells must additionally overcome T cell rejection by the host immune system and toxicities such as graft-versus-host disease^8^. A number of studies have nominated genes that, in principle, can be manipulated to overcome these challenges; however, enacting these strategies in cell products remains a major challenge and will require clinically relevant, robust, nontoxic and multiplexed approaches^9–11^.

CRISPR-based genome editing has become a predominant approach for engineering therapeutic T cell products. CRISPR–Cas9 can facilitate gene inactivation by introducing DNA double-strand breaks (DSBs) or stimulate precise genome editing through homology-directed repair (HDR)^12^. Base editing and prime editing can generate efficient point mutations or small insertions and deletions without introducing DSBs^13,14^. However, each of these approaches results in permanent changes to the genome and potential unintended chromosomal abnormalities induced by on-target or off-target genome editing^15–17^.

An alternative strategy for modulating gene function is through programmable control of endogenous gene expression. We and others have shown that repurposed CRISPR proteins can perturb gene expression in T cells without the formation of DSBs or any permanent change to the genetic code^18–20^. Unfortunately, transcriptional editing approaches such as CRISPRi, CRISPRa and Cas13d require sustained expression of the CRISPR proteins to maintain control of gene expression, which largely precludes their use in therapeutic applications in T cell therapies because of immune recognition of the bacterial Cas proteins and rejection of the transplanted cells^21–23^.

Recent work from our group and others has shown that heritable gene silencing can be achieved through transient expression of epigenetic effectors targeted to specific genomic loci^24–27^. In particular, we showed that CRISPRoff, an epigenetic editor protein composed of dCas9 fused to DNMT3A, DNMT3L and ZNF10 KRAB protein domains, can write an epigenetic silencing program that is persistent for over 450 cell divisions in HEK293T cells^27^ when delivered as plasmid DNA. We also showed that CRISPRoff gene silencing could be reversed by CRISPRon, an epigenetic editor consisting of dCas9 fused to a TET1 catalytic domain that enables targeted erasure of DNA methylation. Importantly, epigenetic editors such as CRISPRoff and CRISPRon do not require DNA damage for their mechanisms of action, thereby eliminating the reliance on specific DNA repair outcomes and the genotoxicity, cytotoxicity and chromosomal abnormalities associated with these pathways. CRISPRoff and CRISPRon epigenome editing (epi-editing) is, thus, in principle, highly multiplexable, making complex therapeutic gene programming efforts possible for a vast array of therapeutic applications. However, this requires efficient and durable epigenetic editing in therapeutically relevant cell types such as primary human T cells.

Here, we develop an optimized, clinically compatible RNA-based epigenetic engineering platform for turning genes on and off in primary human T cells. We show that our T cell epi-editing platform is potent and durable and can be multiplexed, suggesting that this is a versatile approach for broad use in therapeutic applications. CRISPRoff gene silencing is highly specific to the intended targets, is effective in a wide range of therapeutically relevant genes and eliminates the cytotoxicity and chromosomal translocations observed with multiplexed Cas9 gene editing. Additionally, CRISPRon achieves targeted DNA demethylation of an endogenous enhancer, resulting in stable induction of *FOXP3*, a therapeutically relevant gene in human primary T cells. Lastly, we successfully couple targeted epigenome engineering with targeted CAR knock-in (KI) at the *TRAC* locus to generate epi-edited *TRAC* CAR-T cells with enhanced functionality ex vivo and in vivo in a preclinical model of adoptive cell therapy.

## Results

### Durable and specific silencing of endogenous genes in primary human T cells

**Fig. 1 |:**
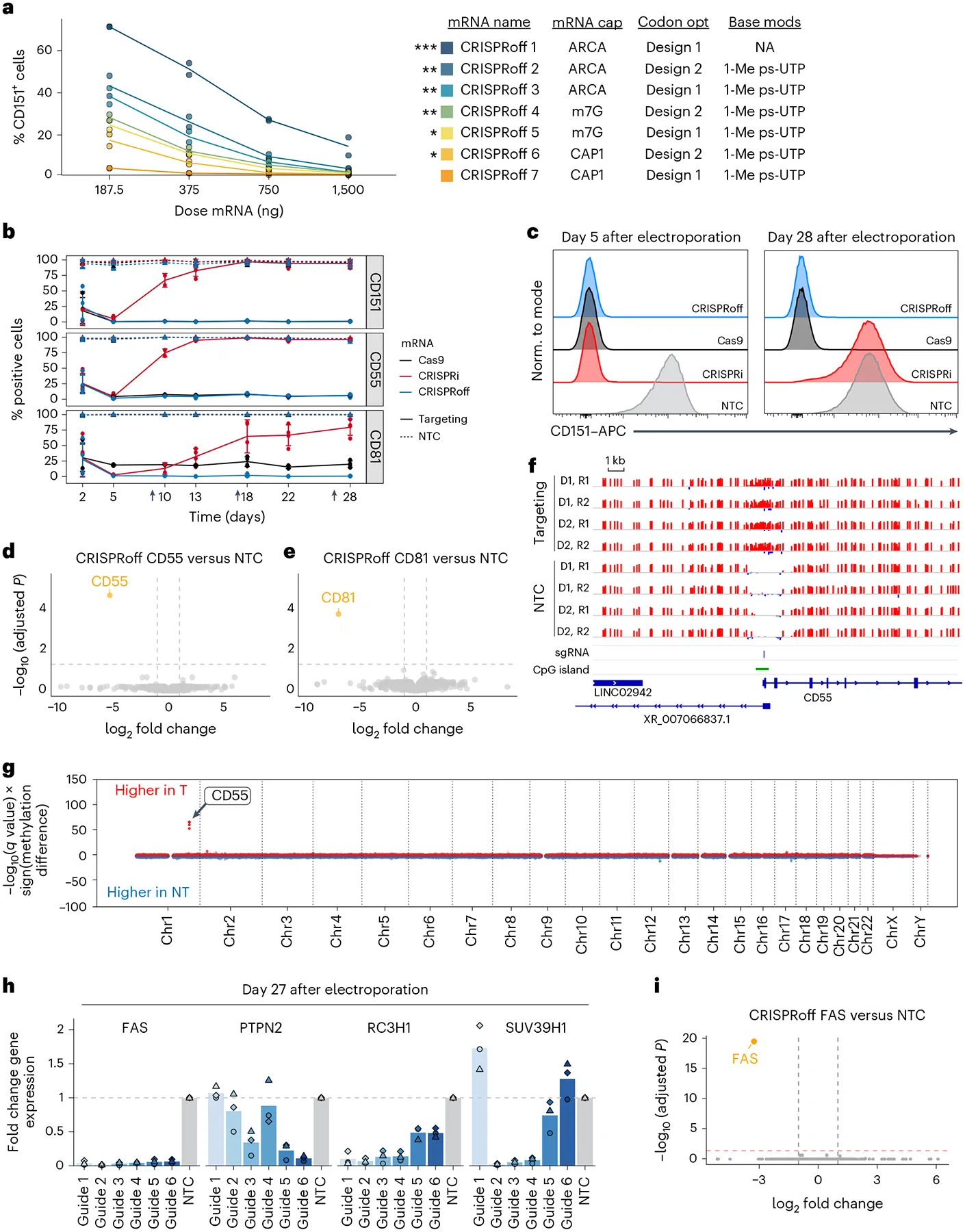
Specific and durable transcriptional silencing by CRISPRoff in primary human T cells. **a**, Comparison of KD efficiency of *CD151* across seven CRISPRoff mRNA designs over a series of mRNA doses. CD151 expression was assessed using flow cytometry 5 days after electroporation. We modeled CD151 positivity as a function of dose and mRNA variant and then computed a *P* value for the difference between CRISPRoff 7 and the rest of the mRNA variants using the standard error (Methods). *P* values were then adjusted using the Benjamini–Hochberg procedure. CRISPRoff 7 was the most potent CRISPRoff mRNA variant as assessed by the degree of *CD151* silencing across CRISPRoff doses (*n* = 2 donors; CRISPRoff 1, ***P = 0.00022; CRISPRoff 2, **P = 0.011; CRISPRoff 3, **P = 0.0096; CRISPRoff 4, **P = 0.001; CRISPRoff 5, **P = 0.0044; CRISPRoff 6, *P = 0.04). **b**, Comparison of Cas9 (black), CRISPRi (red) or CRISPRoff (blue) mRNA KO or KD activity on *CD151*, *CD55* and *CD81* loci over a time course of 28 days after electroporation. Black arrows along the *x* axis indicate restimulations with anti-CD2/CD3/CD28 soluble antibodies (day 9, day 18 and day 27 after electroporation) (*n* = 4 donors except on day 10, where *n* = 2 donors; mean ± s.d.). **c**, Representative flow cytometry histogram plots of *CD151* KD (or KO) by CRISPRoff, CRISPRi or Cas9 on day 5 and day 28 after electroporation. **d**,**e**, Transcriptomic assessment by RNA-seq of CRISPRoff activity and specificity upon silencing of *CD55* (**d**) or *CD81* (**e**) relative to NTC. Cells were electroporated with CRISPRoff mRNA and an sgRNA targeting *CD55* or *CD81* or NTC. Cells were harvested 28 days after electroporation for RNA extraction. Yellow dots indicate significantly downregulated DEGs and gray dots have no significance (empirical Bayes moderated statistics with Benjamini–Hochberg FDR control, adjusted *P* < 0.05; CD55 adjusted *P* = 2.01 × 10^−5^ and CD81 adjusted *P* = 1.65 × 10^−4^; *n* = 2 donors). **f**, Comparison of CpG methylation analyzed by WGBS within a 20-kb window centered on the *CD55* TSS. CGIs are depicted in green and the sgRNA targeting site is annotated. Tracks represent samples electroporated with CRISPRoff mRNA and an sgRNA targeting the *CD55* TSS or NTC for two independent donor replicates (D1 and D2). Cells were collected at 30 days after electroporation. **g**, The Manhattan plot displays DMRs between cells treated with CRISPRoff and an sgRNA targeting *CD55* or NTC and analyzed by WGBS (cells were collected at 30 days after electroporation). Red dots represent DMRs that gained DNA methylation in the targeting sgRNA samples. Blue dots represent DMRs that gained DNA methylation in NTC samples. The arrow denotes the genomic position of *CD55* (*n* = 2 donors performed in technical replicates). **h**, Day 27 transcript levels of *FAS*, *PTPN2*, *RC3H1* (Roquin 1) and *SUV39H1* relative to NTC as measured by RT–qPCR (*n* = 3 donors). **i**, Transcriptomic assessment by RNA-seq of CRISPRoff activity upon silencing of *FAS* relative to NTC. Cells were electroporated with CRISPRoff mRNA and an sgRNA targeting *FAS* or an NTC and then harvested at 7 days after electroporation for RNA extraction. The yellow dot indicates the target gene, which is significantly downregulated, and gray dots have no significance (empirical Bayes moderated statistics with Benjamini–Hochberg FDR control, adjusted *P* < 0.05; FAS adjusted *P* = 3.35 × 10^−20^; *n* = 4 donors).

To determine whether CRISPRoff can stably silence gene expression in human primary T cells, we designed a panel of seven mRNAs encoding the previously reported *Streptococcus pyogenes*-based CRISPRoff-V2.3 effector^27^. We compared the effects of three mRNA cap modifications (Cap1, ARCA and m^7^g), base modifications (1-Me ps-UTP) and two codon optimization algorithms^27,28^ (Supplementary Fig. 1a). The CD151 cell surface protein was selected for initial optimizations given the presence of a known CpG island (CGI) at its promoter and prior validation with CRISPRoff in HEK293T cells^27^. Each mRNA that incorporated base modifications had more *CD151* knockdown (KD) than our unoptimized mRNA, which had no 1-Me ps-UTP substitution (Supplementary Fig. 1b). We also compared codon optimization algorithms (Supplementary Fig. 1c) and mRNA cap structures (Supplementary Fig. 1d). All CRISPRoff mRNA variants demonstrated efficient CD151 KD at high mRNA concentrations, with complete silencing in 85–99% of cells and no observed cellular toxicity (Fig. 1a and Supplementary Fig. 1e). However, dose titration showed significant differences across designs in mRNA potency for gene silencing. CRISPRoff 7 mRNA, which includes ‘design 1’ for codon optimization, the Cap1 mRNA cap and 1-Me ps-UTP substitution, was the most potent design across mRNA concentrations, especially at low mRNA doses. We, therefore, proceeded with CRISPRoff 7 mRNA (referred to as CRISPRoff hereafter) for all subsequent experiments. We then compared CRISPRoff, CRISPRi and Cas9 mRNA activity at four different Lonza 4D nucleofector pulse codes and at four different time points (0, 2, 5 and 12 days after activation) (Supplementary Fig. 1f). Multiple pulse codes performed well for CRISPRoff KD across time points, highlighting the flexibility of this mRNA electroporation approach. We decided to use DS137 for subsequent experiments in part because it has been used previously for mRNA electroporation into T cells^29^. CRISPRoff was sufficiently efficient that we did not need to use any drug selection or cell sorting to select for CRISPRoff-positive cells.

We next examined whether CRISPRoff could initiate and maintain programmable gene silencing in primary human T cells at multiple endogenous gene targets over many cell divisions. We designed experiments to compare CRISPRoff, Cas9 and CRISPRi activity across time when delivered as mRNA to primary human T cells. We selected *CD151*, *CD55* and *CD81* for targeting as all three of these target genes contain known CGIs and are not essential for cell proliferation or survival of T cells in vitro^30^. We previously showed that CRISPRoff and CRISPRi have equivalent design and targeting rules for optimal single guide RNA (sgRNA) activity^27^. Hereafter, throughout this study, for all CRISPRoff and CRISPRi experiments, we used the top predicted 1–6 sgRNAs^31^ without prior validation in T cells. For both CRISPRoff and CRISPRi experiments, we coelectroporated a pool of three sgRNAs targeting within a 250-bp region immediately downstream of the transcription start site (TSS) of each gene or a nontargeting control sgRNA (NTC) along with CRISPRoff or CRISPRi mRNA. For Cas9 experiments, we electroporated one sgRNA predicted for optimal knockout (KO) activity or an NTC along with Cas9 mRNA^32,33^. Cell surface levels of each targeted gene’s protein product were monitored by flow cytometry over a time course of 28 days. As expected, CRISPRi targeting led to transient gene silencing that was progressively lost over time, notably upon T cell restimulation using anti-CD2/CD3/CD28 soluble antibodies on day 9 (Fig. 1b). In contrast, CRISPRoff programmed durable gene silencing that was comparable to Cas9 KO for at least 28 days after electroporation with absence of cell surface expression in over 93% of cells for each gene target (Fig. 1b,c). Notably, CRISPRoff silencing persisted through three anti-CD2/CD3/CD28 soluble antibody restimulations over a 28-day time course, demonstrating that CRISPRoff gene silencing memory is stably propagated across approximately 30–80 cell divisions in vitro (Methods). RNA sequencing (RNA-seq) confirmed that CRISPRoff gene silencing was highly specific, with robust repression of the *CD55* or *CD81* target gene and no other differentially expressed genes (DEGs) at 28 days after electroporation (Fig. 1d,e and Supplementary Fig. 2a). Whole-genome bisulfite sequencing (WGBS) further confirmed the specificity of DNA methylation deposited at the target locus by CRISPRoff; the highest differentially methylated region (DMR) between targeting samples and NTC samples occurred at the *CD55* TSS (Fig. 1f,g).

We then tested the ability to silence therapeutically relevant genes with CGIs that are known to modulate T cell signaling or adoptive T cell function including *FAS* (ref. 34), *PTPN2* (ref. 35), *RC3H1* (Roquin 1)^36^, *SUV39H1* (ref. 37), *MED12* (ref. 38) and *RASA2* (ref. 39). For *FAS*, *PTPN2*, *RC3H1* and *SUV39H1*, we electroporated CRISPRoff mRNA along with the top six predicted sgRNAs for each gene in an arrayed format along-side an NTC and then maintained cells in vitro for up to 27 days after electroporation, with restimulation using anti-CD2/CD3/CD28 soluble antibodies every 9–10 days. We also targeted each gene for KO using Cas9 as described above. Cell pellets were collected for RNA or DNA extraction and bulk RNA-seq (on day 7 after electroporation), indel analysis (on day 7 after electroporation) or qPCR (on day 27 after electroporation) was performed to measure target gene silencing or KO. We found that, for each gene, at least one and generally multiple sgRNAs could potently and durably mediate CRISPRoff silencing of target gene expression (Fig. 1h and Supplementary Fig. 2a). Cas9 gene KO was also efficient (Supplementary Fig. 2b). RNA-seq analysis enabled us to profile the biological consequences of silencing or KO of *FAS*, *MED12*, *PTPN2*, *RC3H1*, *SUV39H1* and *RASA2* and examine the specificity of CRISPRoff gene silencing (Fig. 1I and Supplementary Fig. 2c–n). *FAS* and *RC3H1* were the sole genes decreased upon CRISPRoff targeting of *FAS* and *RC3H1*, respectively. The only genes that decreased upon *SUV39H1* targeting were *SUV39H1* and *LINC02446*; *LINC02446* reduction is almost certainly a biological secondary effect of *SUV39H1* ablation because it was also decreased by *SUV39H1* KO with Cas9 and an independent sgRNA. *PTPN2* targeting also had relatively specific effects on the transcriptome with a modest number of additional downregulated genes, which could be either off-target effects or secondary effects of target KD. *RASA2* KD or *MED12* KD (targeted with a pool of three sgRNAs) had broader effects on the transcriptome. For *MED12*, to further examine CRISPRoff specificity and to determine whether observed DEGs besides the target gene were secondary transcriptional effects or potential off-targets of CRISPRoff, we compared CRISPRoff RNA-seq results to Cas9 KO RNA-seq results (Supplementary Fig. 2n). Many DEGs were shared between *MED12* KD and KO and recapitulate known biology. For example, top downregulated DEGs include *KLF2, CCR7* and *IL7R*, which are all expected biological secondary effects of *MED12* loss on the basis of past observations from our group and others^38,40^ (Supplementary Fig. 2l–n). To further investigate CRISPRoff specificity, we analyzed gene expression changes for neighboring genes within a 100-kb window around each target gene (to look for on-target, off-gene effects). We also examined gene expression changes for predicted off-target sgRNA-binding sites (https://www.idtdna.com/site/order/designtool/index/CRISPR_SEQUENCE) within ±1 kb of a gene’s TSS, according to previously established rules for CRISPRoff activity and specificity^27^. Only one gene across 151 putative off-target sites for all genes targeted showed evidence of potential CRISPRoff off-target activity (Supplementary Fig. 3a–f). Specifically, in *RASA2*-KD samples, we observed decreased expression of *LARP1B*. However, further work would be necessary to determine whether LARP1B is a true off-target or an indirect effect associated with *RASA2* KD. In summary, CRISPRoff gene silencing proved programmable, efficient, specific and durable at many endogenous genes in primary human T cells.

### CRISPRoff silencing at genes that lack CGI annotations

**Fig. 2 |:**
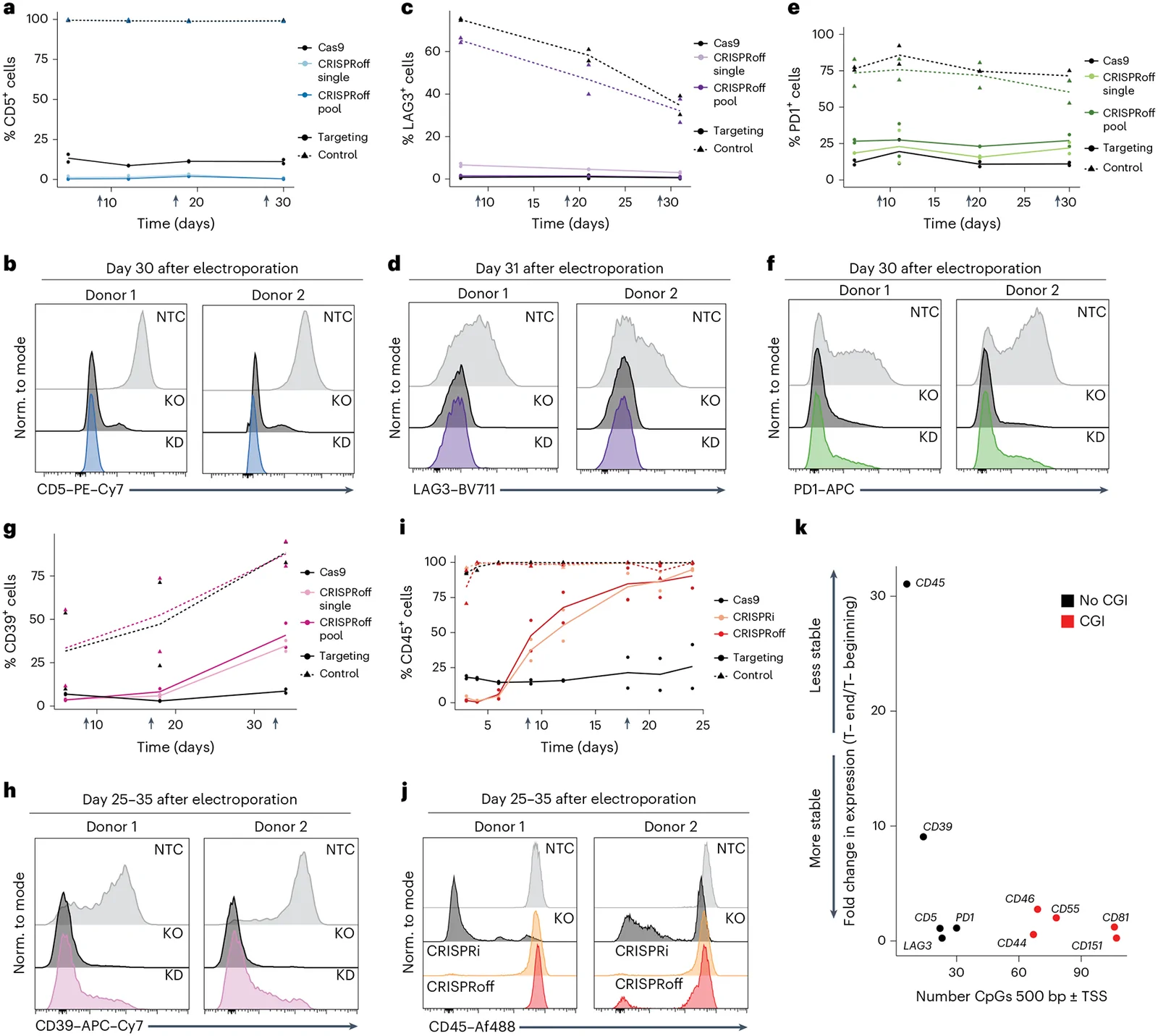
CRISPRoff silencing at genes that lack CGI annotations. **a**, Comparison of Cas9 KO (black) or CRISPRoff KD activity with either a single sgRNA (light blue) or a pool of three sgRNAs (dark blue) at *CD5* over a time course of 30 days after electroporation (*n* = 2 donors). **b**, Representative flow cytometry histogram plots of *CD5* KD (blue) or KO (black) compared to NTC (gray) at 30 days after electroporation. **c**, Comparison of Cas9 KO (black) or CRISPRoff KD activity with either a single sgRNA (light purple) or a pool of three sgRNAs (dark purple) at *LAG3* over a time course of 31 days after electroporation (*n* = 2 donors). **d**, Representative flow cytometry histogram plots of *LAG3* KD (purple) or KO (black) compared to NTC (gray) at 31 days after electroporation. **e**, Comparison of Cas9 KO (black) or CRISPRoff KD activity with either a single sgRNA (light green) or a pool of three sgRNAs (dark green) at *PDCD1* over a time course of 30 days after electroporation (*n* = 2 donors). **f**, Representative flow cytometry histogram plots of *PD1* KD (green) or KO (black) compared to NTC (gray) on day 31 after electroporation. **g**, Comparison of Cas9 KO (black) or CRISPRoff KD activity with either a single sgRNA (light pink) or a pool of three sgRNAs (dark pink) at *CD39* over a time course of 35 days after electroporation (*n* = 2 donors). **h**, Representative flow cytometry histogram plots of *CD39* KD (pink) or KO (black) compared to NTC (gray) at 31 days after electroporation. **i**, Comparison of Cas9 KO (black), CRISPRi (gold) or CRISPRoff activity with a single sgRNA (orange) targeting *CD45* over a time course of 24 days after electroporation (*n* = 2 donors). **j**, Representative flow cytometry histogram plots of KO (black), CRISPRi KD (gold) or CRISPRoff KD (orange) compared to NTC (gray) at 24 days after electroporation. In **a**,**c**,**e**,**g**,**i**, black arrows along the *x* axis indicate restimulations with anti-CD2/CD3/CD28 soluble antibodies. **k**, Fold change in surface protein expression (% positive cells) from the first time point to the last time point for cells treated with CRISPRoff mRNA and the most potent guide targeting the TSS of each respective gene. The number of CpG dinucleotides within ±500 bp of the TSS of each gene is shown on the *x* axis. Red dots indicate genes with CGI annotations in the UCSC genome browser. Black dots indicate genes that do not have a CGI annotation.

In addition to its activity at CGIs, CRISPRoff was previously shown in HEK293T cells to allow durable silencing (in a DNA methylation-dependent manner) of genes lacking a CGI^27^. To explore whether this biology extends to primary T cells, we attempted to use CRISPRoff for durable silencing of five genes lacking CGIs: *CD5*, *LAG3*, *PDCD1*, *ENTPD1* (CD39) and *PTPRC* (CD45). Each of these genes encodes a cell surface protein that is important for sensing external stimuli^41^, signaling^42–44^ and function^45,46^ in T cells. We first electroporated CRISPRoff mRNA and an individual sgRNA or a pool of three sgRNAs targeting the TSS of *CD5*, *LAG3*, *PDCD1* or *CD39* or a single NTC sgRNA. As a control, we also electroporated Cas9 mRNA and one KO sgRNA targeting each of these genes or an NTC sgRNA. We then measured cell surface levels of each target protein at time points up to 35 days after electroporation. For each time point, cells were restimulated using anti-CD2/CD3/CD28 soluble antibodies and cell surface expression was measured 24 h later using flow cytometry. For the non-CGI genes targeted, we observed stable, partially stable or unstable silencing over the course of ~30 days. For *CD5* and *LAG3*, CRISPRoff silencing was comparable to or even more efficient than Cas9 KO. At 30 days after electroporation, the CRISPRoff pooled sgRNA conditions for *CD5* and *LAG3* remained up to 99.5% and 99.1% silenced, respectively (Fig. 2a–d). While we observed some differences between the efficiency of PD1 silencing using CRISPRoff between CD4^+^ and CD8^+^ T cells, PD1 remained stably silenced out to 30 days after electroporation in most cells across of a bulk population (78.15% *PD1*-KD cells versus ~90% *PD1*-KO cells) (Fig. 2e,f and Supplementary Fig. 4). CD39 exhibited partially stable silencing, with a fraction of cells regaining CD39 expression with time, although most cells (53%) remained CD39^−^ negative compared to the NTC on day 35 (Fig. 2g,h). We then evaluated CRISPRoff or CRISPRi mRNA with a single sgRNA targeting the *CD45* TSS or an NTC sgRNA in comparison to Cas9 mRNA with a single sgRNA targeting protein coding exon 2 or an NTC sgRNA. Initially, CRISPRoff, CRISPRi and Cas9 all showed efficient ablation of CD45 (~99% for KD and ~85% for KO); however, by 7 days after electroporation, both CRISPRoff and CRISPRi effects diminished until reaching the levels of the NTC by 24 days after electroporation (Fig. 2i,j). Taken together, we observed a range of how effectively and durably non-CGI genes can be silenced and found that some genes with particularly low levels of CpG dinucleotides around the TSS remain challenging to stably silence (Fig. 2k). Further work is needed to elucidate rules for governing stable silencing at non-CGI genes in primary human T cells. Nonetheless, we demonstrate that expression from CGI and non-CGI genes can be stably silenced in primary human T cells through transient delivery of CRISPRoff.

### Durable multiplexed gene silencing

**Fig. 3 |:**
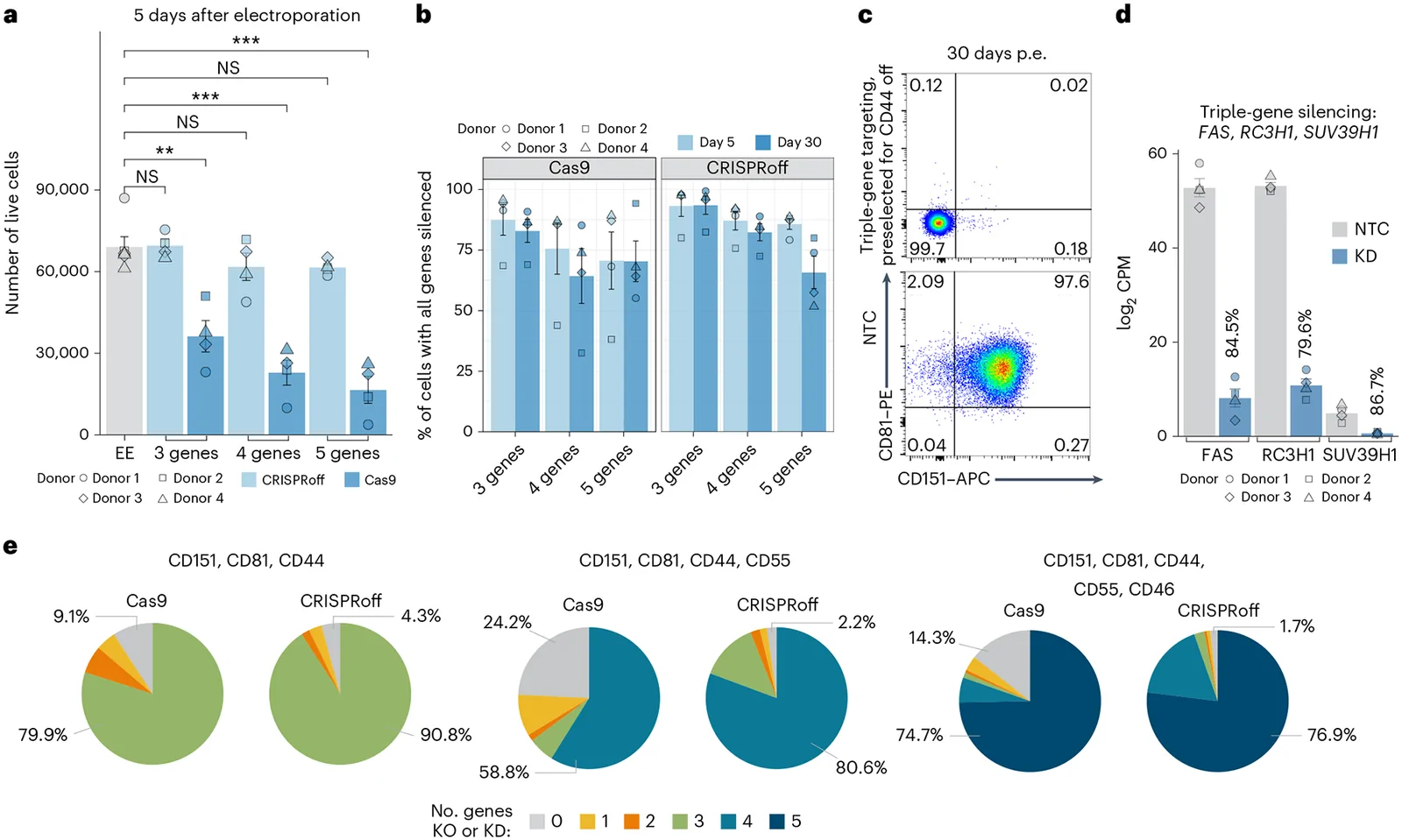
Durable multiplexed gene silencing. **a**, Graph showing the number of live T cells following cell editing by CRISPRoff and Cas9 when targeting either three, four or five genes simultaneously as compared to an empty electroporation (EE) control. Live-cell counts were measured 5 days after electroporation (*n* = 4 donors; mean ± s.e.m.; two-sided Welch’s *t*-test: Cas9, three genes, **P = 0.004; Cas9, four genes, ***P = 0.00015; Cas9, five genes, ***P = 0.00013; NS, not significant). **b**, Plot comparing CRISPRoff versus Cas9 multiplexed gene silencing efficiency targeting three genes (*CD151*, *CD81* and *CD44*), four genes (*CD151*, *CD81*, *CD44* and *CD55*) or five genes (*CD151*, *CD81*, *CD44*, *CD55* and *CD46*) at 5 days and 30 days after electroporation. The percentage of cells with all genes silenced was calculated from flow cytometry analysis (*n* = 4 donors; mean ± s.e.m.). **c**, A representative flow plot of cells targeted for triple-gene (*CD151*, *CD81* and *CD44*) silencing (top) or NTC (bottom). Cells were analyzed at 30 days after electroporation. Top, cells were first gated on CD44-silenced cells and the represented population shows CD81 and CD151 silencing. **d**, An RNA-seq log2 CPM (normalized counts per million) plot showing triple-target-gene KD in cells electroporated with CRISPRoff mRNA and sgRNAs targeting *FAS*, *RC3H1* and *SUV39H1* or an NTC sgRNA. Cells were collected for RNA-seq 7 days after electroporation (*n* = 4 donors; mean ± s.e.m.). **e**, Pie charts depicting the outcomes of three-gene, four-gene or five-gene silencing or KO shown in **b**. Data are representative of one donor.

Epi-editing can modulate gene expression without inducing DSBs, in contrast to Cas9 nuclease targeting. This feature could offer important advantages in the context of multiplexed gene targeting approaches, as genome editing with nuclease-active Cas9 can result in translocations or chromosomal loss, which both have potential to be detrimental to cell proliferation, cell survival and perhaps the safety of therapeutic cell products^9,47–49^. To explore this approach, we simultaneously targeted sets of three, four or five nonessential genes with CRISPRoff or nuclease-active Cas9. Targeting multiple genes with nuclease-active Cas9 resulted in substantial cellular toxicity in human T cells, which may be attributed to the multiple DSBs generated by this approach (Fig. 3a). In contrast, targeting three, four or five genes for silencing with CRISPRoff resulted in minimal to no observable cellular toxicity compared to electroporation alone at either a high or low dose of mRNA (Fig. 3a and Supplementary Fig. 5a,b). CRISPRoff multiplexed epi-editing averaged across four donors was efficient and durable out to 30 days after electroporation with combined silencing of three, four and five target genes at 93.5%, 82.4% and 65.8%, respectively (Fig. 3b,c,e). For some of these multiplexed gene combinations, silencing was marginally improved by increasing the dose CRISPRoff mRNA (Supplementary Fig. 5c–f). For these multiplexing experiments, we confirmed efficient CRISPRoff gene silencing for each gene individually with greater than 95% KD when targeting one sgRNA to the TSS (Supplementary Fig. 5g). We also achieved efficient, multiplexed gene silencing of three potentially therapeutically relevant genes (*FAS*, *RC3H1* and *SUV39H1*), knocking down each target transcript by ~80% (Fig. 3d). We anticipate that durable multiplexing silencing could be further improved by empirically testing individual sgRNAs targeting genes of interest at lower doses to identify sgRNAs with optimized potency for use in multiplexed combinations.

### CRISPRon can target an enhancer region in primary human T cells

**Fig. 4 |:**
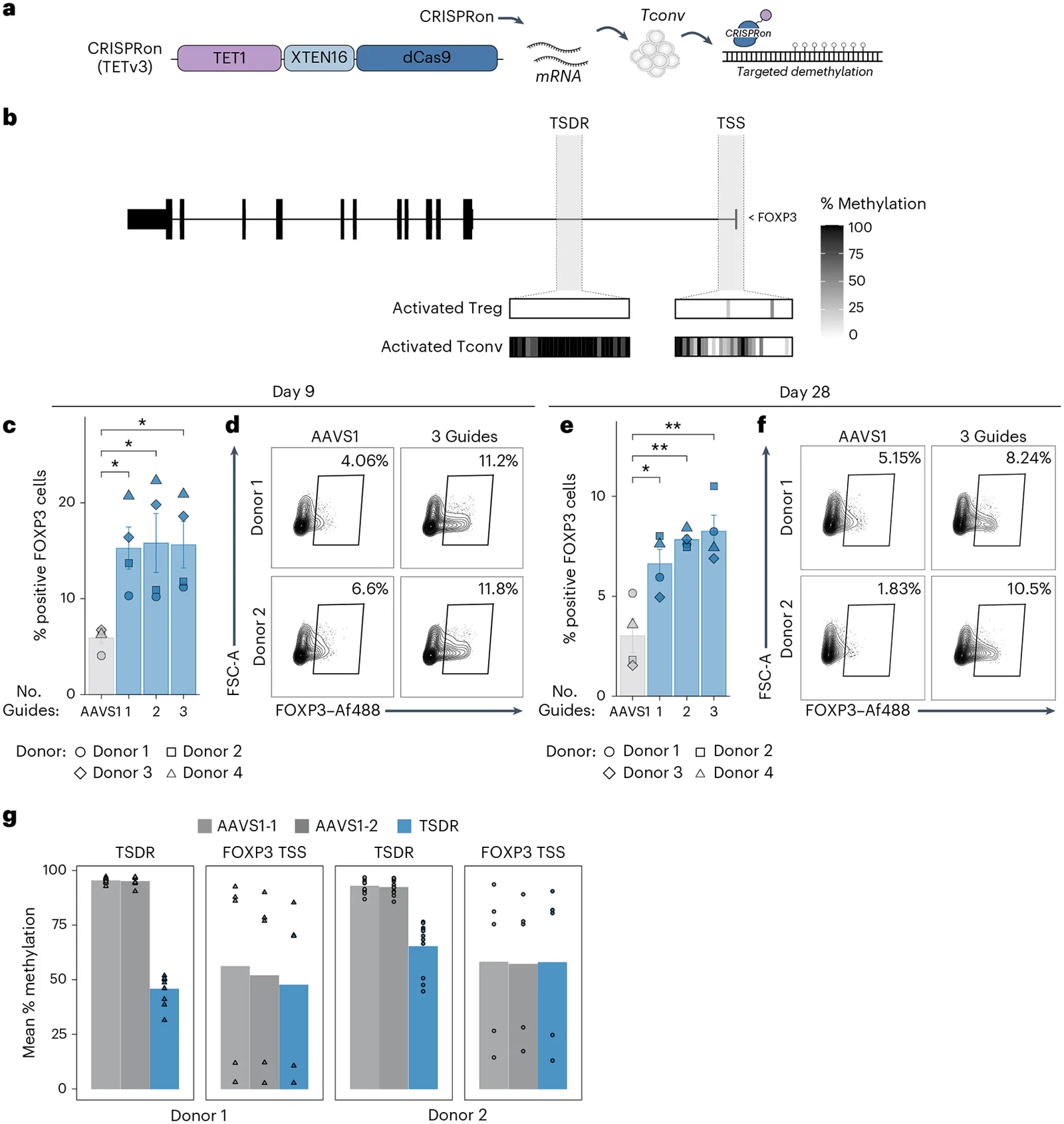
CRISPRon can target enhancer regions in primary human T cells. **a**, Schematic of CRISPRon-TETv3 mRNA, which consists of TET1 catalytic domain fused to dCas9, and work flow of targeted demethylation using mRNA electroporation. **b**, The *FOXP3* gene body with the TSDR and TSS highlighted in gray. The heat map indicates the percent methylation of individual CpGs as measured by PBAT-seq^50^ across the TSDR or TSS between activated Tregs and Tconvs. Each bar in the heat map represents an individual CpG. Data are representative of one donor. **c**, Percentage of FOXP3^+^ Tconv cells after epi-editing with CRISPRon-TETv3 mRNA and 1–3 sgRNAs targeting the TSDR (blue) or AAVS1 control region (gray) as measured by flow cytometry at 9 days after electroporation (*n* = 4 donors per condition; mean ± s.e.m.; two-sided Welch’s *t*-test: for one guide targeting the TSDR, *P = 0.02; for two guides, *P = 0.046; for three guides, *P = 0.024). **d**, Representative flow plots from day 9 after electroporation depicting *FOXP3* expression after epi-editing with CRISPRon-TETv3 targeting the TSDR with a pool of three sgRNAs or an AAVS1 control. **e**, Percentage of FOXP3^+^ Tconv cells after epi-editing with CRISPRon-TETv3 mRNA targeting the TSDR with 1–3 sgRNAs or an AAVS1 control as measured by flow cytometry at 28 days after initial activation (*n* = 4 donors per condition; mean ± s.e.m.; two-sided Welch’s *t*-test: for one guide targeting the TSDR, *P = 0.018; for two guides, **P = 0.0083; for three guides, **P = 0.004). **f**, Representative flow cytometry histograms of FOXP3 median fluorescence intensity for CRISPRon-TETv3 and a pool of three sgRNAs targeting the TSDR or an AAVS1 control at 28 days after electroporation. **g**, Mean percentage of methylation across all CpGs assayed per targeted region (TSDR or TSS). Each triangle (donor 1) or circle (donor 2) represents an individual CpG.

Epi-editing would be further enabled by technology to stably activate target chromatin sites, in addition to the silencing capability demonstrated above. We previously developed CRISPRon, a deactivated Cas9 enzyme fused to the TET1 DNA demethylase catalytic domain, to remove DNA methylation from a targeted locus and induce gene expression^27^. However, this prior work with CRISPRon was performed in immortalized cell lines and only demonstrated reactivation of genes through demethylation of TSSs that were previously silenced by CRISPRoff. Here, we set out to extend the capabilities in three important ways: (1) enable use in primary cells; (2) demonstrate activation of a genomic element that is naturally DNA methylated and epigenetically silenced; and (3) extend the use of epi-editing from TSSs to an enhancer element. We generated three variants of CRISPRon mRNA (TETv3, TETv4 and TETv5), which differed in the linker length used between TET1 and dCas9, with the cap structure and base modifications we optimized for CRISPRoff (Fig. 4a, Supplementary Fig. 6a). To test whether CRISPRon could modulate clinically relevant gene expression in primary human T cells, we turned toward the *FOXP3* (forkhead box P3) locus. FOXP3 is a transcription factor necessary for establishing immune-suppressive regulatory T cells (Tregs). Tregs are essential for immune homeostasis through establishing tolerance against self-antigens, limiting inflammation and aiding in tissue repair. Tregs are usually defined by high and sustained FOXP3 expression, whereas conventional CD4^+^ T cells (Tconvs) only transiently express FOXP3 upon activation. The difference in FOXP3 expression between cell types can be partly attributed to an intronic enhancer that harbors the Treg-specific demethylated region (TSDR), which is completely demethylated in Tregs but remains highly methylated in Tconvs^50^ (Fig. 4b). We hypothesized that targeting the TSDR in Tconvs with CRISPRon could remove endogenous repressive DNA methylation at this regulatory element, resulting in constitutive FOXP3 expression^51^. We isolated CD4 + CD25^low^ Tconvs from two to four human donors and stimulated cells with anti-CD2/CD3/CD28 soluble antibodies. At 2 days after stimulation, we electroporated CRISPRon-TETv3 and five individual sgRNAs targeting the TSDR, *FOXP3* TSS or *AAVS1* safe harbor locus as a control (Supplementary Fig. 6b). We then measured FOXP3 expression at 9 days after initial stimulation, at which point Tconvs have entered a resting state and, thus, should express low amounts of *FOXP3*. Only sgRNAs targeting the TSDR increased *FOXP3* expression, while targeting the TSS did not increase *FOXP3* relative to control sgRNAs (Supplementary Fig. 6b). At 48 h after restimulation, when control Tconvs transiently express *FOXP3*, cells treated with CRISPRon-TETv3 targeting the TSDR expressed higher levels of *FOXP3* than AAVS1-targeted control cells (Supplementary Fig. 6c). We then took the top three performing guides targeting the TSDR from this initial experiment (guide 1, guide 3 and guide 4) and tested them either as individual sgRNAs or as pools of two or three sgRNAs with each CRISPRon mRNA variant. We observed that targeting CRISPRon to the FOXP3 TSDR resulted in an increased fraction of FOXP3^+^ cells across multiple donors, CRISPRon designs and sgRNA number relative to the CRISPRon AAVS1 controls (Fig. 4c,d and Supplementary Fig. 6d).

We maintained the TSDR targeting conditions and AAVS1 control cells in culture to assess the stability and persistence of FOXP3 expression over time, restimulating cells with anti-CD2/CD3/CD28 soluble antibodies every 9–11 days. On day 28 after initial stimulation, Tconvs were collected for flow cytometry and we observed that *FOXP3* expression was stably upregulated in a population of cells over weeks in vitro (Fig. 4e,f and Supplementary Fig. 6e). Targeted bisulfite sequencing confirmed reduced methylation at the TSDR in TSDR-targeted cells even though the bulk bisulfite sequencing was performed on a heterogeneous population of cells, as we did not sort for FOXP3^+^ expression before bisulfite sequencing (Fig. 4g). As expected, methylation at the *FOXP3* TSS did not change when targeting the TSDR (Fig. 4g) nor did it change at other Treg associated genes (*IL2RA* and *IKZF2*) (Supplementary Fig. 6f,g). Our optimized CRISPRon results contrast with a previous effort to demethylate the TSDR locus in a targeted manner in Tconvs, which showed rapid remethylation of the locus at late time points in culture, even when FOXP3^−^ negative clones were isolated^51^. Here, without sorting or clonal isolation, we were able to achieve a significantly increased fraction of FOXP3^+^ cells across donors after 28 days in culture with CRISPRon targeting the TSDR, as compared to the *AAVS1* sgRNA control. These results establish CRISPRon as a powerful tool to control expression of an important endogenous gene expression through enhancer targeting with potential to be applied toward next-generation cell therapies.

### CAR-T cell enhancement with genetic and epigenetic engineering

Having established a robust toolbox for epigenome engineering in primary T cells, we applied it to enhance immune cell therapy function in a preclinical model of cancer. We aimed to use CRISPRoff to enhance CAR-T cell function by simultaneous targeted genomic integration of a CAR (or other antigen receptor) transgene along with targeted epigenetic silencing using CRISPRoff. Targeted insertion of a CAR to the endogenous TCRα constant (*TRAC*) locus using CRISPR–Cas9 genome editing can enhance T cell potency by placing CAR expression under the regulated and dynamic control of the endogenous TCRα promoter, limiting exhaustion and dysfunction^52^. This approach offers potential functional, safety and cost benefits over current lentiviral and gammaretroviral transduction methods. Our rationale for combining this approach with CRISPRoff is based on the clinical observation that introduction of a CAR alone is insufficient to achieve durable responses or cures for most cancers. Our group and many others have identified additional genes that can be disrupted to further enhance CAR-T cell function in challenging tumor microenvironments, which we reasoned would be appropriate targets for epi-editing^38,53–55^. In particular, we discovered that *RASA2* ablation promotes T cell function across a variety of immunosuppressive conditions, improving antigen sensitivity and durable effector function^39^.

**Fig. 5 |:**
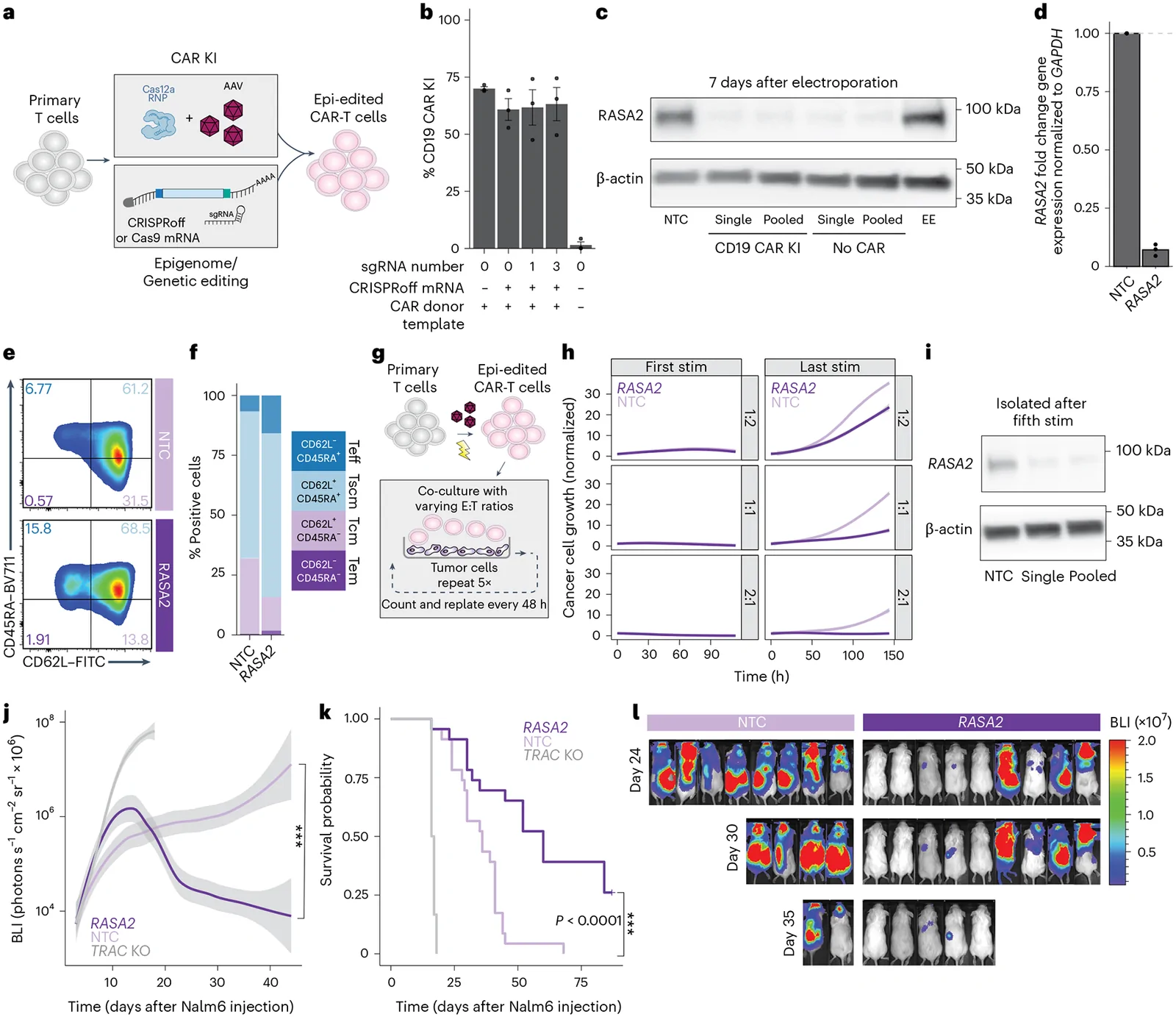
An integrated approach for simultaneous epigenetic and genetic engineering of CAR-T cells. **a**, Schematic of a method for simultaneously generating epigenetically and genetically engineered CAR-T cells using Cas12a RNP for CAR KI. **b**, Graph comparing KI efficiency of CD19-specific CAR with no mRNA present or CRISPRoff mRNA used in combination with an NTC or sgRNA targeting *RASA2*. CRISPRoff was electroporated with either one sgRNA or a pool of three sgRNAs targeting the *RASA2* TSS. Conditions noted as 0 sgRNA indicate an NTC (mean ± s.e.m.; *n* = 3 donors). **c**, Western blot comparison of *RASA2* silencing with CRISPRoff with or without a CD19-specific *TRAC* CAR. CRISPRoff was coelectroporated with either a single NTC sgRNA, a single sgRNA targeting *RASA2* or a pool of three sgRNAs targeting *RASA2*. Data are representative of one donor. **d**, Cells with a *TRAC* CAR KI and *RASA2* KD or NTC were collected at 7 days after electroporation for RT–qPCR. Transcript levels show *RASA2* normalized to *GAPDH*, relative to the NTC (*n* = 3 donors). **e**,**f**, T cell immunophenotypes on day 7 based on CD45RA and CD62L expression, measured by flow cytometry and shown as either raw flow plots (**e**) or bar charts (**f**). Data are representative of one donor. **g**, Schematic of the repetitive stimulation assay to examine the functional efficacy of *RASA2* epi-silenced CAR-T cells. **h**, Graphs show CAR-T cell cytotoxicity according to Incucyte analysis after five repetitive stimulations with target cancer cells. Dark-purple lines indicate *RASA2* epi-edited CAR-T cells and light-purple lines are control-edited CAR-T cells. The line is the mean and shaded areas depict the 95% confidence interval for technical replicates across three independent donors (*n* = 3 donors). Each row represents an E:T ratio (top, 1:2; middle, 1:1; bottom, 2:1). **i**, Western blot for *RASA2* expression in CAR-T cells that were treated with either one sgRNA or a pool of three sgRNAs targeting *RASA2*, which were isolated after the fifth repetitive stimulation. Data are representative of one donor. **j**, NSG mice were injected with 0.5 × 10^6^ Nalm6 cells followed 4 days later by 0.1 × 10^6^ *RASA2* epi-silenced CD19-specific CAR-T cells or CD19-specific CAR-T cells treated with an NTC. Tumor burden was monitored by BLI. The line is the mean and shaded areas depict the 95% confidence interval across replicates (*n* = 4–5 mice per group across four independent experiments and four donors, for a total of 23 mice per group; two-sided Mann–Whitney *U*-test: *P* = 0.0004). Individual experiments are shown in Supplementary Fig. 9. **k**, Survival of *RASA2* epi-silenced CD19-CAR-T cells shown in **i**. Survival curves were compared using a log-rank test (*P* = 2.2 × 10^−16^). **l**, Representative images for the mice shown in **k**.

We reasoned that combining *TRAC* CAR KI with CRISPRoff-mediated silencing of additional targets could boost CAR-T cell function while avoiding translocations and other genotoxic events seen with prior multiplexed KO approaches^56^. We first explored an orthogonal Cas approach using *Acidaminococcus* sp. Cas12a (AsCas12a) ribonucleoproteins (RNPs) for targeted CAR KI in combination with stable epigenetic silencing of RASA2 using the *S*. *pyogenes* dCas9-based CRISPRoff system^57,58^. AsCas12a was precomplexed with a *TRAC* CRISPR RNA (crRNA) and coelectroporated with CRISPRoff mRNA and 1–3 sgRNAs targeting *RASA2*. Following electroporation, cells were transduced with an adeno-associated virus (AAV) HDR template (HDRT) containing a CD19-specific 28z CAR transgene flanked by *TRAC* locus homology arms, which serves as the HDR donor for KI (Fig. 5a). The addition of CRISPRoff mRNA and sgRNAs targeting *RASA2* did not reduce CAR KI efficiency or yield (Fig. 5b). Likewise, CRISPRoff exhibited robust *RASA2* silencing activity, similar in cells with or without integration of a CAR (Fig. 5c,d). In addition, we tested a fully nonviral approach for CAR KI using Cas9-target-site-modified single-stranded DNA (ssDNA) templates that were previously adapted for good manufacturing practice (GMP)^59^. We observed that using the same species of Cas9 and dCas9 for KI and KD resulted in translocations between *TRAC* and *RASA2* and less efficient CAR KI, presumably because of sgRNA swapping that led to Cas9-mediated DSBs at both loci (Supplementary Fig. 7a,b)^60^. To address guide swapping, we tested truncated sgRNAs (16-bp protospacer) for CRISPRoff targeting *RASA2* with the goal of retaining dCas9 binding and transcriptional control while eliminating Cas9 nuclease activity^61^. Truncated sgRNAs ameliorated *RASA2*:*TRAC* translocations (Supplementary Fig. 7a), retained efficient CAR KI (Supplementary Fig. 7b) and maintained silencing activity, albeit to a lesser extent than did full-length sgRNAs (Supplementary Fig. 7c–e). Taken together, we developed multiple approaches that could be made GMP-compatible that combine targeted CRISPR KI and programmable epigenome engineering.

We then tested the durability and functional effect of *RASA2* silencing by CRISPRoff in CAR-T cells through an in vitro repetitive stimulation assay with *RASA2* KD using a pool of three full-length sgRNAs or NTC. Experiments were performed with AsCas12a-based KI using an AAV HDRT given the more stable epigenome engineering observed with the full-length CRISPRoff sgRNAs. Most control CAR^+^ cells displayed an immunophenotype consistent with a T memory stem cell population at 7 days after electroporation on the basis of CD45RA and CD62L expression, although *RASA2*-KD cells shifted slightly to a more T effector-like population, consistent with previous reports^39^ (Fig. 5e,f). *RASA2*-silenced CAR-T cells were cocultured with CD19-expressing tumor cells at multiple effector-to-target (E:T) ratios repeatedly every 48 h (Fig. 5g and Methods). Consistent with previous reports, this repetitive stimulation assay caused control-edited CAR-T cells (treated with CRISPRoff and an NTC sgRNA) to decline progressively in their ability to control cancer cells by the last stimulation (Fig. 5h). *RASA2*-silenced CAR-T cells continued to kill target cells efficiently after five rounds of stimulation (Fig. 5h), consistent with the reported behavior of *RASA2*-KO CAR-T cells^39^. *RASA2* remained stably silenced in RASA2-targeted CAR-T cells isolated after the last stimulation, confirmed by western blot (Fig. 5i). Nonviral Cas9-based KI cells that had *RASA2* silenced with a pool of three truncated sgRNAs also performed better than control-edited cells in a repetitive stimulation assay (Supplementary Fig. 7f).

We next examined the stability of CRISPRoff-induced silencing in CAR-T cells when transferred in vivo. As *RASA2* silencing confers CAR-T cells with an in vivo fitness advantage over control-edited CAR-T cells, we instead chose to target *CD151*, which has no known role in T cell fitness in vivo. First, A375 melanoma cells engineered to express CD19 were engrafted in the flanks of NSG mice. Epi-edited T cells were engineered as previously described with an AsCas12a RNP precomplexed with a *TRAC* crRNA for *CD19*-CAR KI and CRISPRoff mRNA coelectroporated with a pool of three sgRNAs targeting the *CD151* TSS or an NTC (Supplementary Fig. 8a). Epi-edited or control-edited CAR-T cells were cultured in vitro for 1 week after electroporation and then transferred in vivo through tail-vein injection 1 week after A375 engraftment. At 14 days after CAR-T cell transfer, tumors and spleens were isolated from mice and CAR-T cell *CD151* expression was assessed by flow cytometry. Compared to NTC CAR-T cells, *CD151* targeted CAR-T cells obtained from the tumor and spleen retained highly efficient *CD151* KD, suggesting that CRISPRoff silencing is stable upon transfer in vivo and tumor-antigen recognition (Supplementary Fig. 8b–d).

Lastly, we tested the functional efficacy of *RASA2*-silenced CAR-T cells in vivo. As described above, we generated *RASA2*-silenced *TRAC* CAR-T using AsCas12a KI with an AAV template and CRISPRoff with a pool of three full-length sgRNAs targeting RASA2 or a single NTC sgRNA (Supplementary Fig. 9a,b). NSG mice were injected intravenously with Nalm6 leukemia cells and, 4 days later, injected with *RASA2*-silenced *TRAC* CD19–28z CAR-T cells, control *TRAC* CD19–28z CAR-T cells (treated with CRISPRoff and a single NTC sgRNA) or *TRAC*-KO T cells through the tail vein (Supplementary Fig. 9c). Tumor burden was monitored by bioluminescence imaging (BLI) for ~40 days. We found that *RASA2*-silenced CAR-T cells had a significant advantage over NTC CAR-T cells at controlling tumor burden in vivo in cohorts of mice treated with cells from multiple human donors (Fig. 5j and Supplementary Fig. 9d). Mice treated with *RASA2*-silenced *TRAC* CAR-T cells also had significantly extended survival relative NTC *TRAC* CAR-T cells (Fig. 5k,l). Taken together, these data suggest that epi-edited CAR-T cells can maintain stable target gene silencing even through multiple rounds of successful antigen-positive cancer cell killing, enabling functional enhancement of CAR-T cells through silencing of ‘checkpoint’ genes without the need for multiplexed gene cleavage.

## Discussion

We established an all-RNA CRISPR-based epigenetic editing platform for multiplexed primary human T cell programming. Previous work with CRISPRoff and related systems have demonstrated robust and stable epigenetic silencing in cell lines such as HEK293T cells^24,26,27,62^. While important for initial optimization, these cell lines demonstrate a variety of abnormalities such as endogenously low levels of the TET enzymes that reverse DNA methylation from CpG dinucleotides^63^. We now show that, in primary human T cells, which express high levels of TET2 and TET3 enzymes^64^, silencing of endogenous genes with and without a CGI is stable following only transient expression of CRISPRoff^62^. This approach is highly specific to the target loci and durable through multiple T cell activations, numerous cell divisions and transfer in vivo. It is also compatible with massive multiplexing, eliminating the cytotoxicity and genotoxicities observed with genome engineering using nuclease-active Cas9 or base-editing approaches^65^. Critically, because CRISPRon and CRISPRoff need to be delivered only transiently to exert stable effects, they circumvent the immunogenicity of constitutive Cas protein required for altering expression at the RNA level through CRISPRa, CRISPRi or RNA-targeting Cas species^21–23^.

Transient delivery of CRISPRoff is critical to prevent host rejection of the ultimate cellular products. Here, we optimized mRNA delivery for CRISPRoff by combining cap structure, codon optimization and base modifications to substantially increase mRNA potency. This system enables complete silencing of five concurrent targets in this study and we expect this number could be greatly expanded. Our approach is compatible with current electroporation-based manufacturing processes and the required GMP reagents and equipment. We expect that, for any CRISPR technology, there exists the risk of off-targets and this risk should be carefully evaluated for each unique sgRNA and gene target in any clinical program.

Additionally, we developed an all-RNA platform for CRISPRon that can remove endogenous methylation from the TSDR of *FOXP3*. While prior studies with CRISPRon in HEK293T cells have reactivated genes that were previously silenced by CRISPRoff, here, we targeted a critical endogenously methylated noncoding region to establish stable de novo expression of FOXP3 in a CD4^+^ Tconv cell population over time. These data contrast with a previous attempt to deliver plasmid encoding dCas9 fused to TET1 and an sgRNA targeting the TSDR to primary human T cells, which resulted in rapid remethylation of the TSDR over time, even when *FOXP3*-expressing clones were isolated^51^. In this study, we only demonstrated CRISPRon activity at one enhancer; however, future efforts may focus on establishing the generalizability of this tool across diverse genomic elements. We anticipate that multiplexing with both CRISPRon and CRISPRoff will provide a foundation for systematic reprogramming of chromatin architecture in primary human cells.

Lastly, we demonstrated durable silencing for a variety of clinically relevant T cell genes and developed two strategies compatible with clinical translation that combine CRISPRoff silencing of *RASA2* with targeted *TRAC* locus CAR KI, using either truncated sgRNAs or orthogonal Cas species to circumvent the issue of guide swapping. There are many alternative therapeutic targets and a remaining question is how generalizable CRISPRoff-mediated gene silencing will be across different genomic loci with varying amounts of CpG dinucleotides. We show robust and durable silencing at promoter regions both with and without well-defined CGIs. However, silencing at two of the five non-CGI genes exhibited reduced stability. In addition, regulation of gene expression can be complex and driven by multiple regulatory elements in a cell-state-specific manner. Additional studies are needed to establish rules for stable silencing or activation across diverse genomic loci and cell states, as well as the requirements for CpG content and genomic context. We anticipate that large-scale functional genomics screens across promoters, enhancers and other regulatory regions will be enabled by this platform and could shed light on the rules governing stable versus metastable gene silencing. These studies can also provide important information for mapping and dissecting the functions of noncoding elements in the genome, which can lead to novel therapeutic strategies as with the context-specific enhancer targeted in therapies for sickle cell disease and β-thalassemia recently approved by the US Food and Drug Administration^66^. We expect that leveraging CRISPRoff and CRISPRon will offer insights into gene regulation, epigenetic landscapes and the intricacies of cellular differentiation. Moreover, leveraging these technologies in primary human cells paves the way for the next wave of advanced cellular products with finely tuned control of the epigenetic state to improve the potency, durability and safety of engineered cellular therapies.

## Methods

### T cell isolation and culture

Human peripheral blood Leukopaks enriched for peripheral blood mononuclear cells (PBMCs) from deidentified healthy donors were purchased from StemCell Technologies (200–0092). CD3^+^ T cells were isolated using the EasySep Human T cell isolation kit (100–069) per the manufacturer-provided instructions. After isolation, T cells were seeded at 1 × 10^6^ cells per ml and activated with anti-CD3/CD28 Dynabeads (Life Technologies, 40203D). T cells were maintained in culture at a density of 1 × 10^6^ cells per ml throughout and cultured in complete X-VIVO-15 (cX-VIVO), consisting of X-VIVO 15 (Lonza Bioscience, 04–418Q) supplemented with 5% fetal calf serum (R&D systems, lot M19187), 5 ng mL^−1^ IL-7 and 5 ng mL^−1^ IL-15, unless otherwise indicated. CD4^+^CD25^low^ Tconvs were isolated from washed PBMCs using the EasySep Human CD4^+^CD127^low^CD25^+^ Treg isolation kit (StemCell Technologies, 18063) according to manufacturer’s protocol. Tconvs were activated using Immunocult CD2/CD3/CD28 T cell activation reagent (StemCell Technologies, 10990) at 12.5 μl ml^−1^. Tconvs were maintained in culture in cX-VIVO supplemented with 300 IU per ml of IL-2 and passaged every 2–3 days.

### mRNA production

Seven CRISPRoff mRNA products with varying cap structure (m^7^G, Cap1 and ARCA) and codon optimization sequences were purchased from Aldevron and stored at −80 °C. The CRISPRoff 143533B (CRISPRoff v7) mRNA design was used for in vitro transcription (IVT) to make mRNA in house using Cap1 (TriLink Biotechnologies #N-7113–5). For IVT reactions, plasmids containing the CRISPRoff-V2.3 codon-optimized sequence, CRISPRon-TETv3, CRISPRon-TETv4 or CRISPRon-TETv5 were cloned into a mutated T7 promoter plasmid as previously described^67,68^. IVT templates were produced by PCR amplification of CRISPRoff-V2.3 or CRISPRon variants with the forward primer correcting the T7 mutation and reverse primer appending a poly(A) tail, such that the final template contained the wild-type T7 promoter, 5′ untranslated region (UTR) including Kozak sequence, codon-optimized CRISPRoff-V2.3 coding sequence or CRISPRon variants, 3′ UTR and 145-bp poly(A) tail. The PCR product was purified using solid-phase reversible immobilization bead selection and stored at −20 °C until use. IVT reactions were performed with the HiScribe T7 high-yield RNA synthesis kit (New England Biolabs, E2040S) under full substitution of pseudo-UTP and in presence of 4 mM CleanCap AG (which encodes Cap1) (TriLink Biotechnologies, N-7113–5) with the addition of RNAse Inhibitor (New England Biolabs, M0314L) and yeast inorganic pyrophosphatase (New England Biolabs, M2403L). Transcribed mRNA was purified with lithium chloride and eluted in water. After quantification by NanoDrop spectrophotometer and normalization to 1 μg μl^−1^, mRNA product was assessed on an Agilent 4200 TapeStation system and subsequently stored at −80 °C. CleanCap Cas9 mRNA was purchased from TriLink (L-7606).

### Epigenetic or genetic editing with mRNA electroporation

For experiments using Cas9, CRISPRi or CRISPRoff mRNA, fresh CD3^+^ T cells were activated with a 1:1 bead-to-cell ratio with anti-CD3/CD28 Dynabeads (Life Technologies, 40203D) in the presence of 5 ng μl^−1^ IL-7 and 5 ng μl^−1^ IL-15 at 1 × 10^6^ cells per ml. After 2 days of stimulation, T cells were magnetically debeaded, washed with PBS and resuspended in TheraPEAK P3 buffer with supplement (Lonza, G4LP3–126000) at 0.75 × 10^6^ cells in 20 μl. Cas9, CRISPRi and CRISPRoff mRNA were added to 20 μl of cells at an equimolar ratio (1, 1.07 or 1.6 μg, respectively) with 2 μg of chemically modified sgRNA (Synthego) and cells were electroporated on a Lonza 4D Nucleofector using pulse code DS137. Immediately after electroporation, 80 μl of prewarmed cX-VIVO was added to each electroporation well and cells were incubated for 30 min in a CO_2_ incubator at 37 °C followed by the distribution of each electroporation reaction into three wells of a 96-well round-bottom plate. Each well was brought to 200 μl with cX-VIVO. Cells were maintained and expanded by the addition of cX-VIVO every 2 or 3 days and restimulated with ImmunoCult Human CD2/CD3/CD28 T cell activation reagent (StemCell Technologies, 10990) every 9–10 days at 6.25 μl ml^−1^. All sgRNA sequences used are listed in Supplementary Table 1.

To evaluate CRISPRoff mRNA designs, we electroporated seven CRISPRoff mRNA designs across a range of doses along with an sgRNA targeting CD151. We then compared the CRISPRoff activity data across constructs using ordinary least square regression. We modeled CD151 expression as a function of dose and mRNA variant and then computed a *P* value for the difference between mRNA variants across all doses using the standard error. CRISPRoff 7 was the most potent CRISPRoff mRNA variant as assessed by the degree of CD151 silencing across CRISPRoff doses.

For experiments using CRISPRon mRNA, CD4^+^CD25^low^ (Tconv) cells were isolated from PBMCs and activated using Immunocult CD2/CD3/CD28 T cell activation reagent (StemCell Technologies, 10990) at 12.5 μl ml^−1^. Then, 2 days after activation, Tconvs were electroporated with 1.6 μg of CRISPRon mRNA and 2 μg of chemically modified sgRNA (Synthego) with pulse code DS137 as described above. After electroporation, Tconv cells were maintained and expanded in cX-VIVO supplemented with 300 U per ml

### Extracellular and intracellular flow cytometry

For all experiments with flow cytometry as a readout looking at cell surface markers, 0.5 × 10^5^–1 × 10^5^ cells per condition were transferred to a round-bottom 96-well plate, centrifuged, washed once with 200 μl of cell staining buffer and stained with antibodies (1:50 dilution) for 20 min at 4 °C in the dark (antibodies are listed in Supplementary Table 1). Samples were measured using an Attune NXT cytometer with a 96-well autosampler (Invitrogen) and analyzed using FlowJo version 10.9.0 unless otherwise stated. For experiments measuring PD1, LAG3 and CD39 surface expression over time, cells were stimulated with ImmunoCult Human CD2/CD3/CD28 T cell activation reagent (StemCell Technologies, 10990) at 6.25 μl ml^−1^ 24 h before flow cytometry readout. To obtain comparable live-cell counts between conditions, events were recorded using a fixed volume for all samples. To determine the number of cell divisions in in vitro experiments over time, we plated 0.16 × 10^6^ cells into 96-well round-bottom wells following electroporation. We then counted cells on an Attune NXT Cytometer every 48 h or at each passage time.

For intracellular flow cytometry staining, 0.5 × 10^5^–1 × 10^5^ cells per condition were transferred to a 96-well V-bottom plate, centrifuged and washed once with 200 μl of staining buffer. Cells were resuspended in 30 μl of staining buffer containing Ghost Dye red 780 (Tonbo, 13–0865-T500) and antibodies targeting surface proteins of interest and stained for 20 min at 4 °C in the dark. After staining, cells were washed once with 170 μl of staining buffer and then resuspended in 50 μl of 1× FOXP3 Fix/Perm buffer (BioLegend, 421403) and incubated at room temperature for 30 min in the dark. After fixation, cells were permeabilized in 200 μl of 1× FOXP3 Perm buffer for 15 min at room temperature in the dark. After permeabilization, cells were spun down and washed once with 1× FOXP3 Perm buffer and then resuspended in 30 μl of 1× FOXP3 Perm buffer containing antibodies targeting intracellular proteins and stained in the dark at room temperature for 30 min. Following intracellular staining, cells were washed once with the addition of 170 μl of staining buffer and centrifuged at 300*g* for 5 min; the supernatant was removed. Cells were resuspended in 200 μl of staining buffer and then measured using the Attune NXT Cytometer with a 96-well autosampler.

### Bulk RNA-seq

Human primary T cells were harvested 27 days after electroporation (*CD55* and *CD81*) or 7 days after electroporation (*FAS*, *PTPN2*, *RC3H1*, *SUV39H1* and *MED12*). A total of 1 × 10^6^ cells were harvested per condition and RNA was isolated using a Quick-RNA MicroPrep Kit (Zymo, R1050). Isolated RNA was treated with TURBO DNase (Invitrogen, AM2239) and concentrated using an RNA clean and concentrate kit (Zymo, NC0622892). Library preparation was carried out using the QuantSeq FWD V2 with UDI Set A1 kit and UMI module (Lexogen, 191.96). Final libraries were assessed using a 4200 TapeStation (Agilent), quantified using the Qubit ds HS assay kit (Invitrogen) and sequenced as single-end 50-bp reads on a HiSeq 4000 (Illumina) or NextSeq 500 (Illumina).

RNA-seq data were aligned and counts were generated using the RNA-seq pipeline of nf-core (version 3.18)^69^. Raw sequencing reads were quantified using Salmon and summarized to gene-level counts using tximport. Differential gene expression analysis was conducted using the limma-voom framework, with donor variation included as a covariate in the statistical model. Gene expression was normalized using the trimmed mean of *M* values method and lowly expressed genes were filtered before analysis. DEGs were identified by comparing treated samples to NTCs, with significance criteria set at an adjusted *P* value < 0.05 (empirical Bayes moderated statistics with Benjamini–Hochberg correction) and absolute log_2_ fold change > 1. Results were visualized using volcano plots displaying log_2_ fold change versus −log_10_(adjusted *P* value), with genes colored on the basis of significance thresholds or target gene.

The *MED12*-KO RNA-seq data shown in Supplementary Fig. 2n were from previously generated data in the A.M. lab^40^ and are representative of CD4^+^ cells collected 8 days after activation with ImmunoCult Human CD2/CD3/CD28 T cell activation reagent (StemCell Technologies, 10990). Genotyping measured by NGS showed ~80% editing at MED12. The RNA-seq reads were analyzed as previously described and genes with a false discovery rate (FDR)-adjusted *P* value < 0.05 were considered significant. We correlated our *MED12* CRISPRoff KD (76.5% KD) with this dataset to better match the degree of gene disruption as our *MED12* Cas9 KO data only had ~55% indel-editing efficiency (Supplementary Fig. 2b) and we observed fewer DEGs than expected.

Off-target predictions were generated through the Integrated DNA Technology (IDT) CRISPR–Cas9 guide RNA checker (https://www.idtdna.com/site/order/designtool/index/CRISPR_SEQUENCE) for both Cas9 KO and CRISPRoff KD sgRNAs. For CRISPRoff sgRNAs, predicted off-target loci were filtered for sites that fell within ±1 kb of a gene promoter. We also performed ‘on-target, off-gene’ analyses by assessing effects on proximal genes that fell within a 100-kb window around the intended target. Only predicted off-target or proximal genes that had an absolute KD log fold change > 1 and adjusted *P* value < 0.05 were considered potential true off-target genes.

### WGBS

We generated WGBS libraries for 12 samples, corresponding to EE (empty electroporation), NTC and targeting for CD55 across two donors, each done in technical replicate. Genomic DNA was extracted using the QIAamp DNA Mini Kit (Qiagen) and 250 ng of DNA was diluted to 2.27 ng μl^−1^ in 110 μl with 2 μl of 0.5% lambda DNA spike-in and sheared using a Covaris E220 evolution with intensifier for 50 s to an average length of ~500 bp. Sonicated DNA was recovered using the MinElute reaction cleanup kit (Qiagen), bisulfite conversion was performed using the EZ DNA methylation-Gold kit (Zymogen) and the resulting ssDNA was quantified on the Qubit ssDNA assay kit (Invitrogen). Library preparation was performed using the xGen methylation-sequencing DNA library preparation kit (IDT, 10009860) and xGen Normalase UDI primers plate 1 (IDT, 10009796). The prepared libraries were quantified on a 4200 TapeStation system (Agilent) and Qubit double-stranded DNA HS assay kit (Invitrogen). Libraries were sequenced using paired-end 150-bp reads on a NovaSeqX with a 10% PhiX spike-in to diversify the sample pools.

Raw WGBS-seq FASTQ files were processed using the nf-methylseq: 2.6.0 pipeline^69^ with the default parameters along with the ‘--three_prime_clip_R1 10’ and ‘--three_prime_clip_r2 10’ options. Differential CpG DNA methylation analysis was performed using the methylKit R package^70^. CpG methylation data from Bismark coverage files was imported. To search for differentially methylated tiles, the ‘tileMethyl-Counts’ function was used with options ‘win.size = 1000’ and ‘step. size = 100’. DMRs were scored by the percentage methylation difference and *q* values were calculated using the ‘calculateDiffMeth’ function with ‘overdispersion = MN’ and ‘adjust = BH’ options using the replicates as a covariate in fitting the model. Results were visualized as Manhattan plots to display −log_10_-transformed *P* values associated with individual methylation tiling windows. Statistically significant DMRs with FDR < 0.05 (Benjamini–Hochberg) were colored on the basis of their methylation status. To visualize the methylation status at individual loci in Integrative Genomics Viewer (IGV), the base-level methylation status was extracted from BedGraph files from the nf-methylseq pipeline. Then, results were converted into an IGV-friendly format and data were displayed as bar charts, in which methylated regions were considered as a methylation percentage of 50–100% shown in the range of 0.5 to 1 in red and unmethylated regions were considered as a methylation percentage of 0–50% shown in the range of −1 to −0.5 in blue.

### PBAT-seq visualization

PBAT-seq files at the *FOXP3* locus were provided^50^. PBAT-seq analysis was conducted as previously described^50^. PBAT-seq tracks were visualized using a sliding binning strategy with a bin size of 1,500 and step size of 300 in ggplot2 (version 3.5.1).

### Targeted bisulfite sequencing

A total of 200,000 cells were collected for conditions coelectroporated with CRISPRon and Guide 3 targeting the TSDR or two AAVS1 control targets, spun down and frozen at −80 °C. Targeted bisulfite sequencing was conducted by EpigenDX at two sites across the *FOXP3* locus (TSDR or TSS) and off-target sites including *IL2RA* and *IKZF2*.

### Lysis, RNA extraction and reverse transcription (RT) for qPCR

Cells were lysed and reverse-transcribed as described below. Briefly, 0.1 × 10^5^–0.2 × 10^5^ T cells were spun down in 96-well U-bottom plates and washed once with DPBS (without Ca^2+^ and Mg^2+^) (StemCell Technologies, 37350). Cells pellets were either frozen on dry ice and then stored at −80 °C until further use or lysed in 50 μl of complete RNA lysis buffer (9.6 mM Tris-HCl (pH 7.8), 3 U per ml proteinase K, 300 U per ml DNAse 1, 0.5 mM MgCl_2_, 0.44 mM CaCl_2_, 10 μM DTT and 0.1% (w/v) Triton X-114). Cells were incubated in RNA lysis buffer for 8 min at room temperature and then 30 μl of lysed cells were added to 3 μl of RNA stop solution in a new 96-well PCR plate (1 mM proteinase K inhibitor, 90 mM EGTA and 113 μM DTT in UltraPure water) and incubated for 3 min at room temperature to stop the lysis reaction. Then, 32 μl of RevertAid RT kit (Thermo Fisher, K1691) was aliquoted in a separate 96-well PCR plate and 8 μl of the lysis samples were added and mixed. RT was performed in a thermocycler with samples incubated at 25 °C for 10 min, 37 °C for 60 min and 95 °C for 5 min. Samples were either immediately used for qPCR or frozen and stored at −80 °C.

A master mix was made using TaqMan Fast advanced master mix for qPCR (Thermo Fisher, 4444557) and primer probes (IDT) that targeted either the housekeeping gene, *GAPDH*, or a gene of interest (*FAS*, *MED12*, *PTPN2*, *RASA2*, *RC3H1* or *SUV39H1*). The final concentration of primers was 0.5 μM and that of probes was 0.25 μM. Next, 15 μl of master mix was added to 9.6 μl of complementary DNA from the RT reaction above and qPCR was performed in 5-μl reactions with technical quadruplicates in a 384-well plate format using the QuantStudio real-time PCR system (Thermo Fisher). To analyze the data, the *C*_*t*_ values of the technical quadruplicates were first averaged and then the Δ*C*_*t*_ was calculated by subtracting the *GAPDH* housekeeping *C*_*t*_ value from the averaged experimental values. The ΔΔ*C*_*t*_ was then calculated from subtracting the Δ*C*_*t*_ of the NTC from the ΔC_*t*_ of the experimental samples. The fold change in gene expression was then calculated (2^−ΔΔ*Ct*^).

### Multiplex editing with CRISPRoff in T cells

T cells were electroporated as described above. In multiplexed conditions with either CRISPRoff or Cas9 mRNA, each gene targeted received 1.6 μg of sgRNA. Cells were prepared for flow cytometry to collect live-cell counts and cell surface protein expression as described above. FCS files were analyzed using FlowJo (version 10.9.0) to create a gating scheme; cells were gated on lymphocytes, then single cells and then live cells and a positive or negative gate was drawn for each target gene. To calculate the proportion of cells with a given number of knocked down genes, the FlowJo workspace was read into R using the ‘flowCore’, ‘CytoML’ and ‘openCyto’ packages. First, each individual cell was recorded as either positive or negative for each target gene, with negative indicating KD of a target, on the basis of thresholds set in FlowJo. These thresholds were verified through visualization with the ‘ggcyto’ R package. Each cell was then annotated with the total number of genes successfully knocked down, from zero to five target genes. Lastly, the proportion of all cells for each number of knocked down genes was calculated.

### Epi-edited CAR-T cell production

For nonviral integration of a BCMA-specific CAR transgene at the *TRAC* locus, T cells were isolated and stimulated as described above. After 48 h of stimulation, cells were magnetically debeaded and prepared for electroporation. Briefly, to prepare the guide RNA targeting the *TRAC* locus, aliquots of crRNA and *trans*-activating crRNA (Edit-R, Dharmacon Horizon) were thawed and mixed 1:1 (v/v) and annealed by incubation at 37 °C for 30 min to form an 80 μM solution. ssDNAenh was mixed into the gRNA solution at a 0.8:1 volume ratio before adding 40 μM Cas9-NLS (Berkely QB3 MacroLab) at a 1:1 (v/v) to attain a molar ratio of sgRNA-Cas9 of 2:1. Final RNP mixtures were incubated at 37 °C for 15–30 min, after which 50 pmol of RNP was used for each electroporation. The *TRAC*-targeting Cas9-RNP was mixed with a 2,923-nt ssDNA HDRt encoding the BCMA-specific CAR transgene, incubated for 10–15 min and then mixed with cells that were resuspended in 20 μl of TheraPEAK P3 buffer with supplement (Lonza, G4LP3–126000). The CRISPRoff mRNA and sgRNA targeting *RASA2* or NTC (Synthego) were added on top of the cells last and then cells were electroporated using the Lonza 4D Nucleofector with pulse code EH115. For any experiments incorporating an RNP in the electroporation and mRNA, we used pulse code EH115, as this code is the most effective for RNPs (demonstrated elsewhere) while still maintaining efficiency for mRNAs. For *RASA2* silencing with CRISPRoff mRNA, we codelivered either a chemically modified full-length (20 nt) sgRNA or a chemically modified truncated sgRNA (17 nt with the last base pair mismatched to effectively make a 16-nt truncated guide) (Synthego).

For integration of a CD19-specific CAR transgene at the *TRAC* locus using AAV6, Alt-R A.s. Cas12a (Cpf1) Ultra (IDT, 10001272) was mixed with a crRNA targeting the TRAC locus (IDT) at room temperature for 10–15 min. Cells that were resuspended in 20 μl of TheraPEAK P3 buffer with supplement (Lonza, G4LP3–126000) were mixed with *TRAC*-Cas12a-RNP and CRISPRoff mRNA and sgRNA targeting either *RASA2* or an NTC were added on top of the cells and electroporated using pulse code EH115. At 30 min after electroporation, cells were transduced with AAV encoding the CD19-CAR as previously described^52^. The AAV-ITR plasmids containing the 1928z CAR transgene and *TRAC*-targeting homology arms for HDR was packaged into AAV6 by transfection of HEK293T cells together with pHelper and pAAV Rep-Cap plasmids using polyethylenimine. AAVs were further purified using iodixanol gradient ultracentrifugation. AAVs were tittered using qPCR on DNase I (New England Biolabs)-treated, proteinase K (Qiagen)-digested samples. qPCR was performed with SsoFast Eva-Green Supermix (BioRad, 1725201) on a StepOnePlus real-time PCR System (Applied Biosystems). AAV was added to the cells at a multiplicity of infection of 1 × 10^5^ and cells were incubated overnight in serum-free medium. Then, 1 day after electroporation, the AAV-containing medium was removed and the edited T cells were resuspended in fresh cX-VIVO and expanded using standard culturing conditions. The KI efficiency for both nonviral-mediated HDRT and AAV HDRT KI was evaluated by flow cytometry several days later.

### Digital droplet PCR (ddPCR)

Genomic DNA from 1 × 10^6^–2 × 10^6^ cells was purified using the QIAamp DNA mini kit (Qiagen) following the manufacturer’s protocol. DNA was quantified using the NanoDrop One (Thermo Fisher Scientific). All DNA samples were digested with HindIII in 10× rCutSmart buffer (New England Biolabs) before the ddPCR. A ddPCR assay was designed to measure the occurrence of balanced translocations between *TRAC* and *RASA2*. The assays used a pair of primers targeting a balanced translocation with TRAC on the 5′ end and RASA2 on the 3′ end and a fluorescent FAM probe. A pair of primers targeting the housekeeping gene *RPP30* were included as a reference using a fluorescent HEX probe. The percentage of the translocation occurrences was calculated on the basis of the number of FAM^+^ droplets normalized to the HEX^+^ droplets.

ddPCR was performed using a QX200 ddPCR system (BioRad) following the manufacturer’s protocols. The reaction mix consisted of ddPCR Supermix for probes (no dUTP; BioRad), 900 nM of each primer, 300 nM of the FAM probe, 450 nM of the HEX probe and 400 ng of purified, digested genomic DNA. A 20-μl PCR reaction was used to generate lipid droplets with an automated droplet generator (BioRad). PCR amplification was performed using the following conditions: 95 °C for 5 min and 42 cycles of 94 °C for 30 s (ramp: 2.5 °C s^−1^) and 62 °C for 1 min (ramp: 2.5 °C s^−1^), followed by an enzyme deactivation at 98 °C for 5 min. Readout was performed with QX200 droplet reader (BioRad) and ddPCR droplet reader oil (BioRad). Data analysis was conducted with the QX manager software (BioRad) and thresholds were set manually to obtain the number of positive droplets for each channel.

### Western blotting

For immunoblotting experiments, 2 × 10^6^–3 × 10^6^ cells were harvested, resuspended in 70 μl of Pierce radioimmunoprecipitation assay buffer (Thermo Fisher, 89901) supplemented with protease and phosphatase inhibitor cocktail (Fisher Scientific, 78440) and incubated at 4 °C for 40 min. The protein concentrations were determined using the Qubit protein and protein broad-range assay kits (Invitrogen, Q33211). Then, 15 μg of protein per sample was loaded onto 4–15% Tris–glycine SDS gels (BioRad) followed by transfer to PVDF membrane (BioRad) using the Biorad Trans-Blot transfer system. After transfer, membranes were blocked with 5% (w/v) nonfat milk in PBS containing 0.1% Tween-20 for 30 min. Primary antibody incubations were performed for either 2 h at room temperature or overnight at 4 °C (antibodies provided in Supplementary Table 1).

### In vitro repetitive stimulation assay

For in vitro cytotoxicity assays, we generated epi-silenced CAR-T cells with either *TRAC* BCMA-specific CAR KI using our nonviral approach or *TRAC* CD19-specific CAR-T cells using AAV as described above. For coculture assays, we generated CD19^+^ or BCMA^+^ nuclear-localized RFP^+^ A375 melanoma target cells. At 6 days after electroporation, 300 of these target cells were seeded in 50 μl of complete RPMI per well in a 384-well plate. Complete RMPI includes RPMI (Gibco, 21870076), 10% fetal calf serum (R&D systems, lot M19187), 1% L-glutamine, 1% penicillin–streptomycin, 10 mM HEPES solution (Sigma, H0887) and 1 mM sodium pyruvate (Gibco, 11-360-0*7*0). The next morning, epi-silenced *TRAC* CD19-specific CAR-T cells or BCMA-CAR-T cells were counted and CAR expression was assessed by flow cytometry. CAR-T cell numbers were normalized and added to the target cells according to the indicated E:T cell ratios. The final per-well volume was 100 μl. Target cell counts were measured using the Incucyte live-cell imaging system (Sartorius) with imaging at 6-h intervals based on RFP expression.

For repetitive stimulation assays, CD19-A375 or BCMA-A375 target cells were seeded in complete RPMI medium 1 day before coculture. The next day, half of the medium was replaced with cX-VIVO and CD19-CAR-T cells or BCMA-CAR-T cells were seeded on top of the target cells at a 1:1 E:T ratio. This was repeated every 48 h for up to 5–7 stimulations. For each coculture, CAR-T cells were collected, strained through a 70-μm filter and counted using an Attune NXT Cytometer (Invitrogen). CAR expression was assessed using flow cytometry before each repetitive stimulation to normalize CAR-T cell counts between conditions. Before using the CAR-T cells for any downstream assay, the T cells were collected, counted and purified using EasySep Release human CD45 positive selection kit (StemCell, 100–0105).

### Heritability of CRISPRoff-induced silencing in CAR-T cells in vivo

All mice for animal experiments were housed and used in accordance with ethical guidelines approved by the University of California, San Francisco (UCSF) Institutional Animal Care and Use Committee (IACUC). All animal experiments were performed with 8–12-week-old female NOD-*scid IL2rg*^−/−^ (NSG) mice were purchased from Jax. To assess whether CRISPRoff-mediated silencing persists in CAR-T cells in vivo, we generated epi-edited Cas12a-compatible *TRAC* CD19-CAR-T cells in combination with CRISPRoff mRNA and a pool of three sgRNAs targeting *CD151* or an NTC. Mice were injected with 1 × 10^6^ A375 melanoma cells (engineered to express CD19) through subcutaneous injection to the right flank. Then, 1 week later, mice were randomized on the basis of width and length of the tumors and 7.5 × 10^5^ epi-edited or control-edited CAR-T cells were injected into the tail vein. Mouse health and tumor growth were monitored over time. At 14 days after CAR-T cell injection, mice were humanely killed and tumors and spleens were isolated and prepared for flow cytometry.

### CD19-epi-silenced CAR-T cells and Nalm6 xenograft model

We generated Cas12a-compatible *TRAC* CD19-CAR-T cells treated with CRISPRoff mRNA and three guides targeting *RASA2* or an NTC as described previously. Mice were intravenously injected with 0.5 × 10^6^ FFluc–GFP NALM6 cells and then, 4 days later, injected with 0.1 × 10^6^ *RASA2*-epi-silenced CD19-CAR-T cells or control-edited CD19-CAR-T cells. CRISPRoff silencing activity of *RASA2* in CAR-T cells was validated using western blot or RT–qPCR before injection. If *RASA2* silencing was not observed in CD19-CAR-T cells before injection (because of electroporation error), we excluded those conditions from analysis. Tumor burden was monitored using BLI over time and weight was assessed as were any signs of morbidity per our UCSF IACUC protocol guidelines. For all experiments, mice were randomized on the basis of the BLI signal from day 3 after Nalm6 injection to ensure equal tumor distribution in each group before T cells were transferred.

## Supplementary Material

Supplementary Table

Supplementary Figures

The online version contains supplementary material available at https://doi.org/10.1038/s41587-025-02856-w.

## Data Availability

The data discussed in the publication were deposited to the National Center for Biotechnology Information Gene Expression Omnibus under accession number GSE306915 (RNA-seq) and GSE306917 (WGBS). Source data are provided with this paper.

## Code availability

Code used in this paper is available through Github (https://github.com/GilbertLabUCSF/T_Cell_CRISPRoff/).
