# Supplementary material for: Integrated epigenetic and genetic programming of primary human T cells
Source: Nat Biotechnol. 2025 Oct 21;44(9):1470–81. doi: 10.1038/s41587-025-02856-w (PMC13336058; doi:10.1038/s41587-025-02856-w)

nature biotechnology

Supplementary information https://doi.org/10.1038/s41587-025-02856-w

# Integrated epigenetic and genetic programming of primary human T cells

In the format provided by the authors and unedited

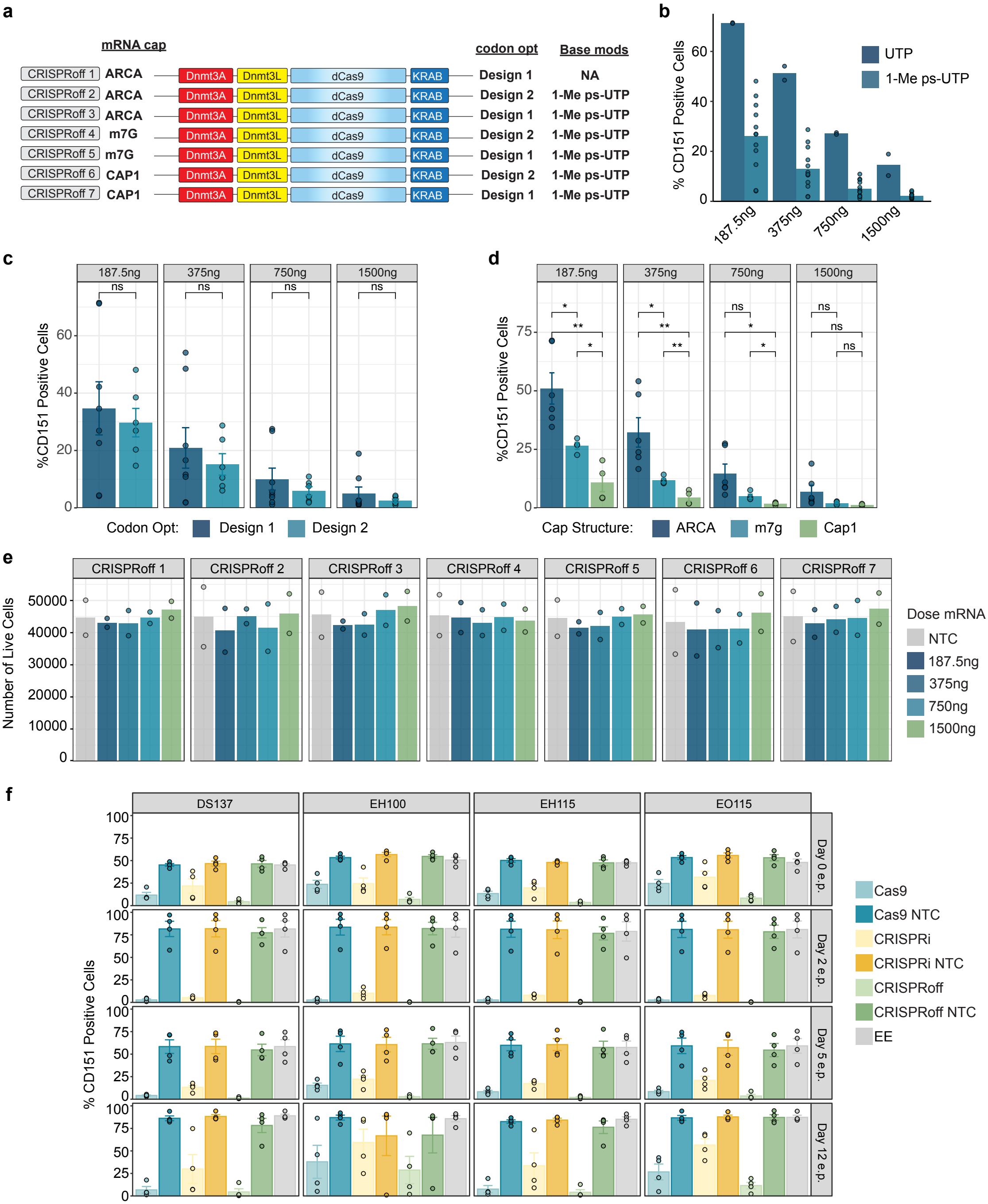
a
mRNA cap
codon opt
Base mods
CRISPRoff 1 ARCA Dnmt3A Dnmt3L dCas9 KRAB Design 1 NA
CRISPRoff 2 ARCA Dnmt3A Dnmt3L dCas9 KRAB Design 2 1-Me ps-UTP
CRISPRoff 3 ARCA Dnmt3A Dnmt3L dCas9 KRAB Design 1 1-Me ps-UTP
CRISPRoff 4 m7G Dnmt3A Dnmt3L dCas9 KRAB Design 2 1-Me ps-UTP
CRISPRoff 5 m7G Dnmt3A Dnmt3L dCas9 KRAB Design 1 1-Me ps-UTP
CRISPRoff 6 CAP1 Dnmt3A Dnmt3L dCas9 KRAB Design 2 1-Me ps-UTP
CRISPRoff 7 CAP1 Dnmt3A Dnmt3L dCas9 KRAB Design 1 1-Me ps-UTP
b
% CD151 Positive Cells
UTP
1-Me ps-UTP
187.5ng
375ng
750ng
1500ng
c
187.5ng
375ng
750ng
1500ng
ns
%CD151 Positive Cells
Codon Opt: Design 1 Design 2
d
187.5ng
375ng
750ng
1500ng
%CD151 Positive Cells
Cap Structure: ARCA m7g Cap1
e
CRISPRoff 1
CRISPRoff 2
CRISPRoff 3
CRISPRoff 4
CRISPRoff 5
CRISPRoff 6
CRISPRoff 7
Number of Live Cells
Dose mRNA
NTC
187.5ng
375ng
750ng
1500ng
f
DS137
EH100
EH115
EO115
Day 0 e.p.
Day 2 e.p.
Day 5 e.p.
Day 12 e.p.
% CD151 Positive Cells
Cas9
Cas9 NTC
CRISPRi
CRISPRi NTC
CRISPRoff
CRISPRoff NTC
EE

**Supplemental Figure 1: Optimization of CRISPRoff mRNA for durable silencing in primary human T cells. (A)** Schematic of seven CRISPRoff mRNA variants tested in primary human T cells. **(B)** Comparison of CD151 KD efficiency at 4 days post-electroporation between CRISPRoff mRNAs that incorporated base modifications (1-Me ps-UTP) versus standard UTP at 4 doses of mRNA. Each dot represents an mRNA variant across two donors. **(C)** Comparison of CD151 KD efficiency at 4 days post-electroporation between codon optimization strategies for dCas9 (Design 1 versus Design 2) at four doses of mRNA. Each dot represents an mRNA variant across two donors ($n$ = 2 donors, mean ± s.e.m.; two-sided Welch's $t$-test). **(D)** Comparison of CD151 KD efficiency at 4 days post-electroporation between mRNA cap structures (ARCA, m7G, Cap1). Quantification of CD151 KD is depicted as fold change relative to the unoptimized mRNA design (CRISPRoff 1). Each dot represents an mRNA variant across two donors (mean ± s.e.m.; $^{*}P < 0.05$ and $^{**}P < 0.01$, two-sided Welch's $t$-test) **(E)** Live cell counts collected at four days post-electroporation for each of the CRISPRoff mRNA variants ($n$ = 2 donors). **(F)** Four individual Lonza pulse codes (DS137, EH100, EH115, and EO115) were tested for mRNA electroporation of either Cas9, CRISPRi, or CRISPRoff mRNA at four separate time points, either before initial activation (Day 0) or after initial activation (2 days post-activation, 5 days post-activation, or 12 days post-activation) with anti-CD3/CD28 Dynabeads. Cells were harvested five days after each electroporation timepoint and CD151 protein expression was quantified by flow cytometry ($n$ = 4 donors, mean ± s.e.m.).

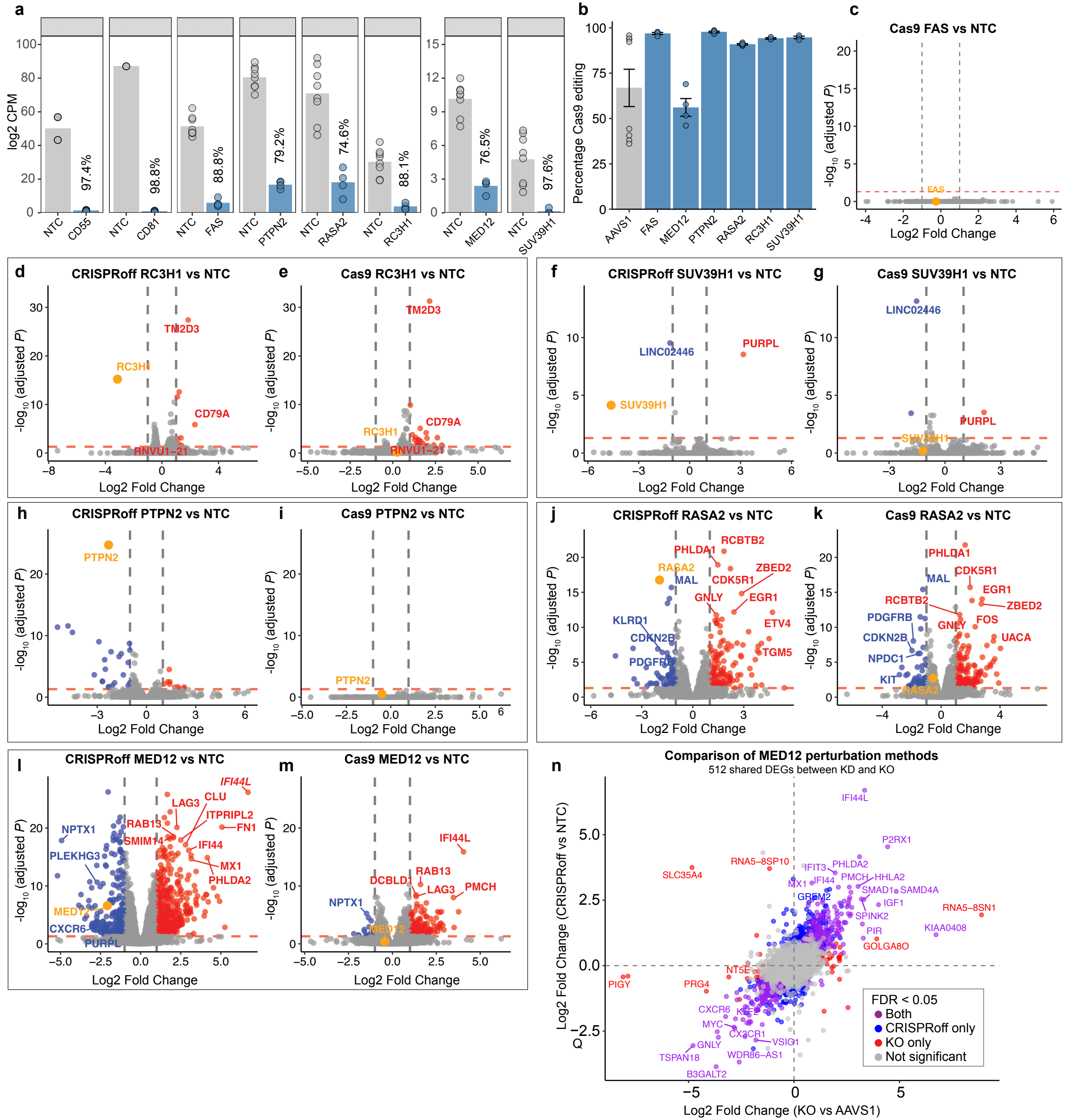

a
log2 CPM
NTC
CD55
97.4%
CD81
98.8%
FAS
88.8%
PTPN2
79.2%
RASA2
74.6%
RC3H1
88.1%
MED12
76.5%
SUV39H1
97.6%
b
Percentage Cas9 editing
AAVS1
FAS
MED12
PTPN2
RASA2
RC3H1
SUV39H1
c
Cas9 FAS vs NTC
d
CRISPRoff RC3H1 vs NTC
e
Cas9 RC3H1 vs NTC
f
CRISPRoff SUV39H1 vs NTC
g
Cas9 SUV39H1 vs NTC
h
CRISPRoff PTPN2 vs NTC
i
Cas9 PTPN2 vs NTC
j
CRISPRoff RASA2 vs NTC
k
Cas9 RASA2 vs NTC
l
CRISPRoff MED12 vs NTC
m
Cas9 MED12 vs NTC
-log10 (adjusted P)
Log2 Fold Change
n
Comparison of MED12 perturbation methods
512 shared DEGs between KD and KO
Log2 Fold Change (CRISPRoff vs NTC)
Log2 Fold Change (KO vs AAVS1)
FDR < 0.05
Both
CRISPRoff only
KO only
Not significant

**Supplemental Figure 2: Durable at specific CRISPRoff silencing at therapeutically relevant genes in T cells. (A)** An RNA-sequencing log2 CPM (normalized counts per million) plot showing cells electroporated with CRISPRoff and sgRNAs targeting *CD55*, *CD81*, *FAS*, *PTPN2*, *RASA2*, *RC3H1*, *MED12*, or *SUV39H1* as compared to NTC treated cells. The percent KD is shown for each gene. (CD55 and CD81 were collected 27 days post-electroporation; $n$ = 2 donors) (FAS, PTPN2, RASA2, RC3H1, MED12, and SUV39H1 were collected on day 7 post-electroporation; $n$ = 4 donors). **(B)** Nuclease-active Cas9 editing efficiency at each of the therapeutically relevant target loci collected for RNA-seq as measured by TIDE indel analysis. **(C-M)**Transcriptomic assessment by RNA-seq of CRISPRoff or Cas9 activity upon silencing or KO, respectively, of **(C)** *FAS*, **(D-E)** *RC3H1*, **(F-G)** *SUV39H1*, **(H-I)** *PTPN2* or **(J-K)** *RASA2* or **(L-M)** *MED12* relative to NTC. For CRISPRoff treated samples, cells were electroporated with the most potent guide identified from RT-qPCR for each gene in panel Figure 1H. For both Cas9 and CRISPRoff treated samples, cells were harvested for RNA extraction and bulk RNA sequencing seven days post-electroporation ($n$ = 4 donors). The target gene for each panel is labeled in yellow, blue dots indicate significantly downregulated DEGs, red dots indicate significantly upregulated DEGs, and grey dots have no significance (Empirical-Bayes moderated statistics with Benjamini–Hochberg FDR control, adjusted $P$ < 0.05). **(N)** Comparison of gene $\log_2$-fold change between CRISPRoff and CRISPR KO targeting *MED12*. CRISPR KO for MED12 RNA-sequencing data is from Arce et al [40]. Non-significant hits are shown in grey and significant hits are colored by direction of effect between experiments (FDR < 0.05) (CRISPR KO data: $n$ = 2 donors. CRISPRoff data: $n$ = 4 donors).

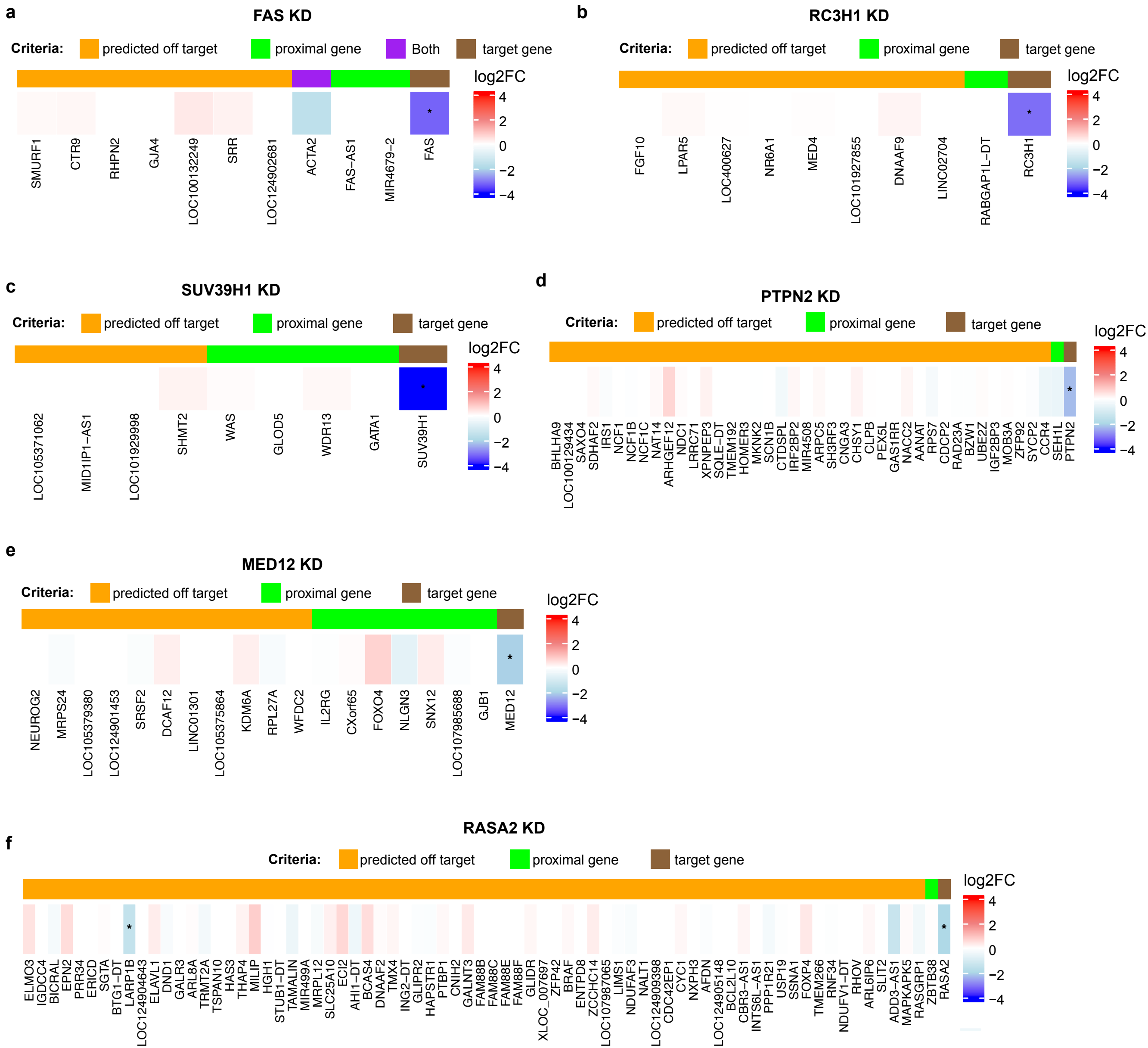
a
FAS KD
Criteria: predicted off target proximal gene Both target gene
log2FC
4 2 0 −2 −4
SMURF1 CTR9 RHPN2 GJA4 LOC100132249 SRR LOC124902681 ACTA2 FAS−AS1 MIR4679−2 FAS
b
RC3H1 KD
Criteria: predicted off target proximal gene target gene
log2FC
FGF10 LPAR5 LOC400627 NR6A1 MED4 LOC101927855 DNAAF9 LINC02704 RABGAP1L−DT RC3H1
c
SUV39H1 KD
Criteria: predicted off target proximal gene target gene
log2FC
LOC105371062 MID1IP1−AS1 LOC101929998 SHMT2 WAS GLOD5 WDR13 GATA1 SUV39H1
d
PTPN2 KD
Criteria: predicted off target proximal gene target gene
log2FC
BHLHA9 LOC100129434 SAXO4 SDHAF2 IRS1 NCF1 NCF1B NCF1C NAT14 ARHGEF12 NDC1 LRRC71 XPNPEP3 SQLE−DT TMEM192 HOMER3 MKNK2 SCN1B CTDSPL IRF2BP2 MIR4508 ARPC5 SH3RF3 CNGA3 CHSY1 CLPB PEX5L GAS1RR NACC2 AANAT RPS7 CDCP2 RAD23A BZW1 UBE2Z IGF2BP3 MOB3A ZFP92 SYCP2 CCR4 SEH1L PTPN2
e
MED12 KD
Criteria: predicted off target proximal gene target gene
log2FC
NEUROG2 MRPS24 LOC105379380 LOC124901453 SRSF2 DCAF12 LINC01301 LOC105375864 KDM6A RPL27A WFDC2 IL2RG CXorf65 FOXO4 NLGN3 SNX12 LOC107985688 GJB1 MED12
f
RASA2 KD
Criteria: predicted off target proximal gene target gene
log2FC
ELMO3 IGDCC4 BICRAL EPN2 PRR34 ERICD SGTA BTG1−DT LARP1B LOC124904643 ELAVL1 DND1 GALR3 ARL8A TRMT2A TSPAN10 HAS3 THAP4 MILIP HGH1 STUB1−DT TAMALIN MIR499A MRPL12 SLC25A10 ECI2 AHI1−DT BCAS4 DNAAF2 TMX4 ING2−DT GLIPR2 HAPSTR1 PTBP1 CNIH2 GALNT3 FAM88B FAM88C FAM88E FAM88F GLIDR XLOC_007697 ZFP42 BRAF ENTPD8 ZCCHC14 LOC107987065 LIMS1 NDUFAF3 NALT1 LOC124909398 CDC42EP1 CYC1 NXPH3 AFDN LOC124905148 BCL2L10 CBR3−AS1 INTS6L−AS1 PPP1R21 USP19 SSNA1 FOXP4 TMEM266 RNF34 NDUFV1−DT RHOV ARL6IP6 SLIT2 ADD3−AS1 MAPKAPK5 RASGRP1 ZBTB38 RASA2

**Supplemental Figure 3: Off-target assessment of CRISPRoff. (A-F)** Off-target assessment of CRISPRoff silencing as measured by RNA-seq when targeting **(A)** *FAS*, **(B)** *RC3H1*, or **(C)** *SUV39H1*, **(D)** *PTPN2*, **(E)** *MED12*, or **(F)** *RASA2*. Predicted off-target sites were generated through IDT's CRISPR-Cas9 guide RNA design checker for each of the CRISPRoff KD guides, and loci were filtered for ±1kb around a gene promoter. Proximal genes within 100kb of the target gene were also assessed. Genes are ordered by left to right by highest to lowest off-target score generated from IDT. Predicted off-target genes that met significance thresholds (adjusted $P > 0.05$ and absolute log2 fold change > 1) are denoted with a star ($n = 4$ donors). Predicted off-targets are annotated with an orange box, proximal genes are annotated with a green box, and the target gene is annotated with a brown box.

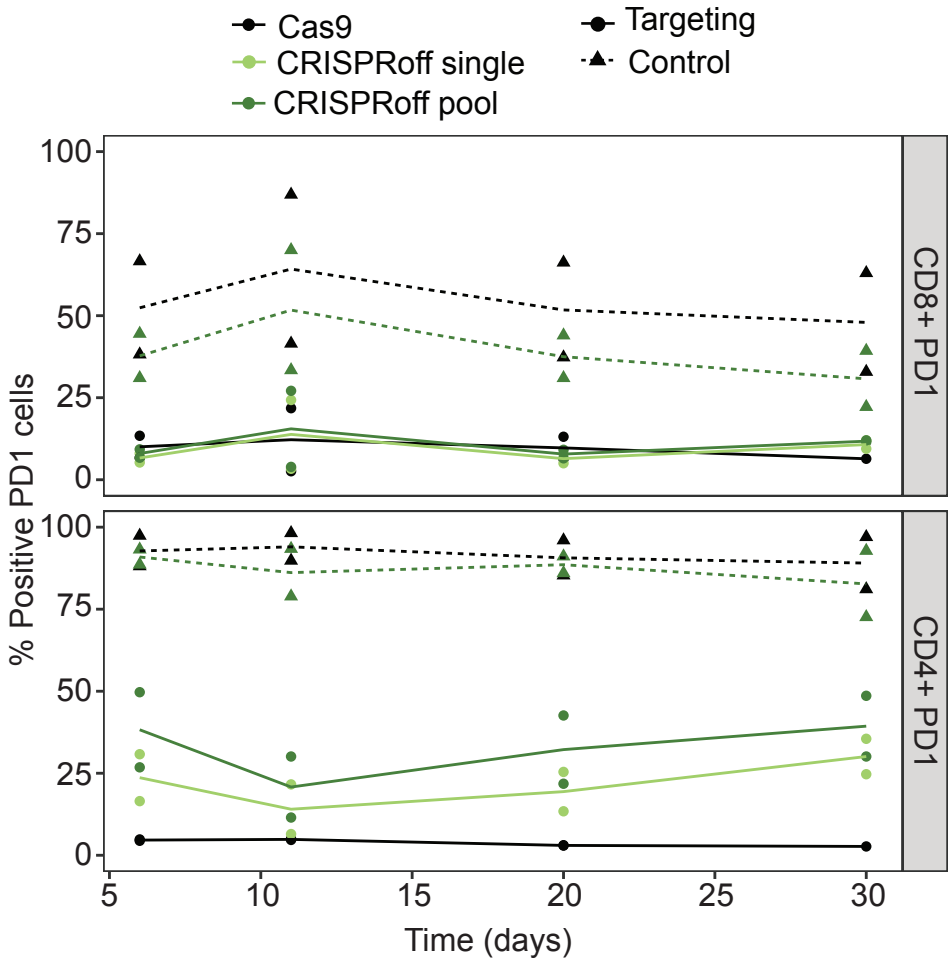
Cas9
CRISPRoff single
CRISPRoff pool
Targeting
Control
CD8+ PD1
CD4+ PD1
% Positive PD1 cells
Time (days)
100
75
50
25
0
5
10
15
20
25
30

**Supplemental Figure 4: CRISPRoff silencing of PD1 in CD8$^+$ and CD4$^+$ T cells.** Comparison of Cas9 KO (black) or CRISPRoff KD activity with either a single sgRNA (light green) or a pool of three sgRNAs (dark green) at *PDCD1* over a time course of 30 days post-electroporation. CD8$^+$ T cells are shown in the top graph, and CD4$^+$ T cells are shown in the bottom graph. Cells were restimulated with anti-CD2/CD3/CD28 at 24 hours prior to each flow cytometry timepoint (*n* = 2 donors).

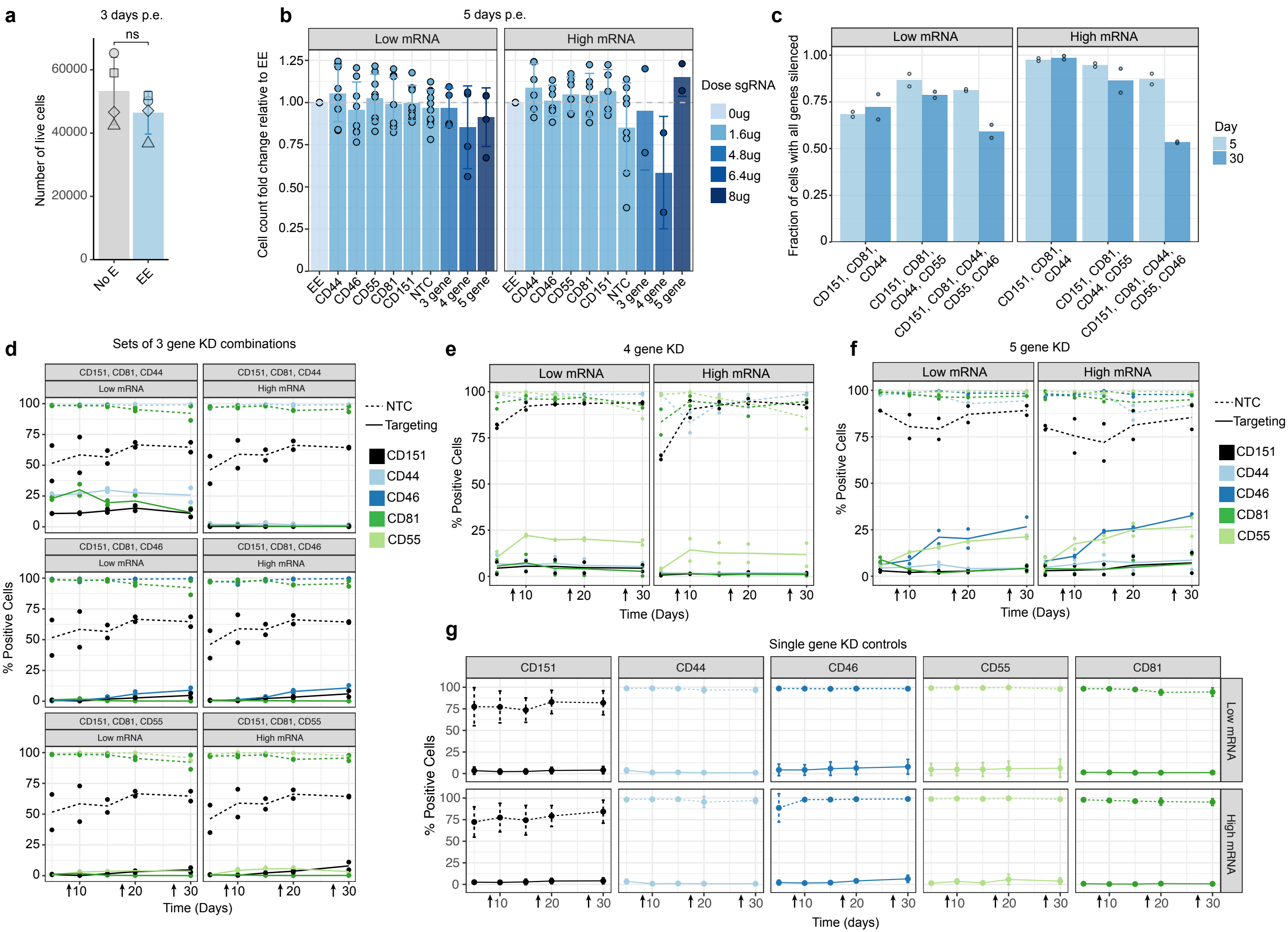

a
3 days p.e.
ns
Number of live cells
No E
EE
b
5 days p.e.
Cell count fold change relative to EE
Low mRNA
High mRNA
EE
CD44
CD46
CD55
CD81
CD151
NTC
3 gene
4 gene
5 gene
Dose sgRNA
0ug
1.6ug
4.8ug
6.4ug
8ug
c
Fraction of cells with all genes silenced
Low mRNA
High mRNA
CD151, CD81, CD44
CD151, CD81, CD44, CD55
CD151, CD81, CD44, CD55, CD46
Day
5
30
d
Sets of 3 gene KD combinations
CD151, CD81, CD44
CD151, CD81, CD46
CD151, CD81, CD55
Low mRNA
High mRNA
NTC
Targeting
CD151
CD44
CD46
CD81
CD55
% Positive Cells
Time (Days)
e
4 gene KD
Low mRNA
High mRNA
% Positive Cells
Time (Days)
f
5 gene KD
Low mRNA
High mRNA
NTC
Targeting
CD151
CD44
CD46
CD81
CD55
% Positive Cells
Time (Days)
g
Single gene KD controls
CD151
CD44
CD46
CD55
CD81
Low mRNA
High mRNA
% Positive Cells
Time (days)

**Supplemental Figure 5: Durable multiplexed gene silencing with CRISPRoff. (A)** Viability of cells that were either not electroporated (No E; grey bar) or empty electroporated (EE; blue bar). Empty electroporated cells were treated with P3 buffer only. (*n* = 4 donors per condition, mean ± SD; two-sided Welch's *t*-test). **(B)** Viability of cells electroporated with CRISPRoff at two doses of mRNA (low = 1.5 µg, high = 3 µg). Cells were co-electroporated with either one sgRNA targeting a single gene (sgRNA dose = 1.6 µg), three genes simultaneously (4.8 µg sgRNA), four genes simultaneously (6.4 µg of sgRNA) or five genes simultaneously (8 µg sgRNA). For multiplexed conditions, each gene target received one sgRNA to the TSS. Live cell counts were collected at 5 days post-electroporation, and cell counts were normalized and displayed as fold-change relative to an empty electroporation condition. (*n* = 8 donors for single gene targeting, NTC, and EE; *n* = 4 donors for multiplex conditions in the low mRNA condition; *n* = 2 donors for multiplex conditions in the high mRNA condition, mean ± SD) **(C)** Comparison of three-gene, four-gene, and five-gene multiplex silencing with CRISPRoff at either a high or low dose of mRNA. Flow cytometry data were collected at 5 days post-electroporation and 30 days post-electroporation, and the percentage of cells with all genes silenced for a given multiplex combination was calculated (see Methods) (*n* = 2 donors). **(D)** Multiplex analysis of triple-gene CRISPRoff silencing when targeting three distinct combinations of genes (*CD151*, *CD81*, *CD44*), (*CD151*, *CD81*, *CD46*), (*CD151*, *CD81*, *CD55*) over a timecourse of 30 days post-electroporation with CRISPRoff mRNA and the indicated sgRNAs. NTC are shown as dotted lines, and targeting sgRNAs are shown as solid lines. Cells were restimulated every nine days post-initial activation, indicated by black arrows (*n* = 2 donors). **(E,F)** Multiplex analysis of either four-gene **(E)** or five-gene **(F)** CRISPRoff gene silencing over a timecourse of 30 days post-electroporation with CRISPRoff mRNA and the indicated sgRNAs. NTC are shown as dotted lines, and targeting sgRNAs are shown as solid lines. Cells were restimulated every nine days after initial activation, indicated by black arrows (*n* = 2 donors). **(G)** The graphs show CRISPRoff silencing of individual genes (*CD151*, *CD44*, *CD46*, *CD55*, or *CD81*) over a timecourse of 30 days post-electroporation of CRISPRoff mRNA and the indicated sgRNA. Cells were re-stimulated every nine days after initial activation with anti-CD2/CD3/CD28 soluble antibodies. Black arrows indicate restimulation timepoints. NTC are shown as dotted lines and targeting sgRNAs are shown as solid lines (mean ± SD, *n* = 10 donors).

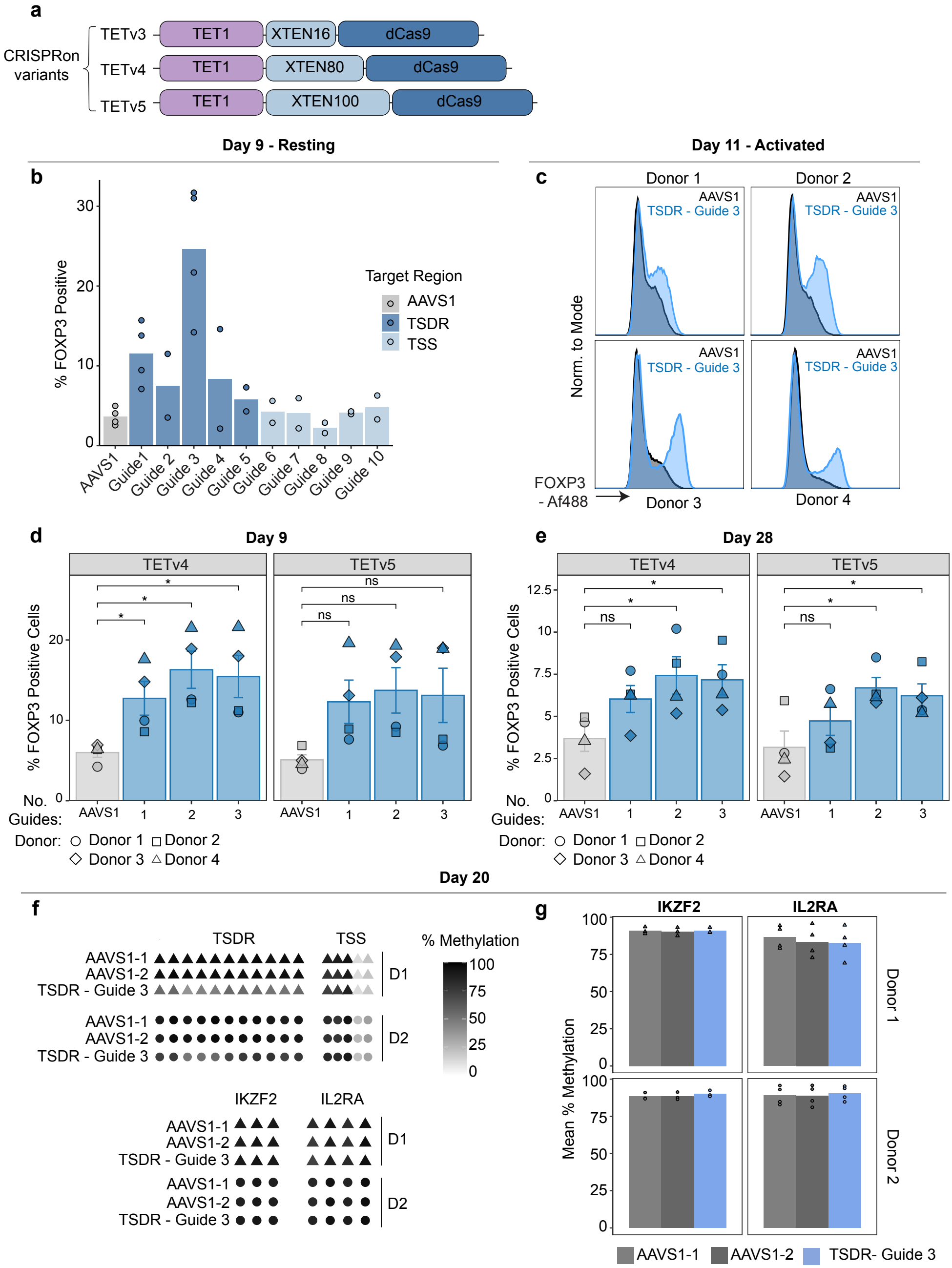
a
CRISPRon variants
TETv3
TET1
XTEN16
dCas9
TETv4
TET1
XTEN80
dCas9
TETv5
TET1
XTEN100
dCas9
Day 9 - Resting
Day 11 - Activated
b
% FOXP3 Positive
Target Region
AAVS1
TSDR
TSS
AAVS1
Guide1
Guide 2
Guide 3
Guide 4
Guide 5
Guide 6
Guide 7
Guide 8
Guide 9
Guide 10
c
Donor 1
Donor 2
Donor 3
Donor 4
AAVS1
TSDR - Guide 3
Norm. to Mode
FOXP3 - Af488
d
Day 9
TETv4
TETv5
% FOXP3 Positive Cells
No. Guides:
Donor: Donor 1 Donor 2 Donor 3 Donor 4
e
Day 28
TETv4
TETv5
% FOXP3 Positive Cells
No. Guides:
Donor: Donor 1 Donor 2 Donor 3 Donor 4
Day 20
f
TSDR
TSS
% Methylation
AAVS1-1
AAVS1-2
TSDR - Guide 3
D1
D2
IKZF2
IL2RA
g
IKZF2
IL2RA
Mean % Methylation
Donor 1
Donor 2
AAVS1-1
AAVS1-2
TSDR- Guide 3

**Supplemental Figure 6: Targeting the *FOXP3* TSDR with CRISPRon in primary human T cells. (A)** Schematic of three CRISPRon variants (TETv3, TETv4, and TETv5), each of which consists of TET1 catalytic domain fused to dCas9 with varying XTEN linker lengths. **(B)** Percentage of FOXP3 expressing cells after Tconv received CRISPRon mRNA-TETv3 and sgRNAs targeting the TSDR (dark blue), the TSS (light blue), or AAVS1 control region (grey) as measured by flow cytometry at nine days post-electroporation (AAVS1 controls and Guides 1 & 3 targeting the TSDR: $n$ = 4 donors. For the remaining conditions: $n$ = 2 donors). **(C)** Flow cytometry histograms showing FOXP3 expression in cells treated with CRISPRon-TETv3 mRNA and Guide 3 targeting the *FOXP3* TSDR or an AAVS1 control sgRNA at 48 hours after restimulation with anti-human CD2/CD3/CD28 soluble antibodies. **(D-E)** Percentage of $FOXP3^+$ Tconv cells after epi-editing with CRISPRon mRNA variants (TETv4, TETv5) and 1-3 sgRNAs targeting the TSDR (blue) or AAVS1 control region (grey) as measured by flow cytometry at 9 days post-electroporation **(D)** or 28 days post-electroporation **(E)**. Cells treated with a single sgRNA received Guide 3, cells treated with a pool of two sgRNAs received Guide 1 and Guide 3, and cells treated with a pool of three sgRNAs received Guide 1, Guide 3, and Guide 4. ($n$ = 4 donors per condition, mean ± s.e.m.; two-sided Welch's $t$-test; For cells collected on day 9 treated with TETv4: 1 guide targeting the TSDR $^*P$ = 0.044, 2 guides $^*P$ = 0.018, 3 guides $^*P$ = 0.033. For cells collected on day 28 treated with TETv4: 2 guides targeting the TSDR $^*P$ = 0.037, 3 guides $^*P$ = 0.026. For cells collected on day 28 treated with TETv5: 2 guides targeting the TSDR $^*P$ = 0.027, 3 guides $^*P$ = 0.046). **(F)** Targeted bisulfite sequencing analysis indicates the percent methylation of individual CpGs assayed within either the TSDR, the FOXP3 TSS, *IKZF2* TSS or *IL2RA* TSS for cells treated with CRISPRon mRNA and Guide 3 targeting the *FOXP3* TSDR or an AAVS1 control. Each triangle (donor 1) or circle (donor 2) representants an individual CpG. **(G)** The mean percentage methylation across the *IKZF2* or *IL2RA* regions shown in **(F)** when cells were treated with CRISPRon mRNA and Guide 3 targeting the *FOXP3* TSDR or an AAVS1 control. Each triangle (donor 1) or circle (donor 2) represents an individual CpG.

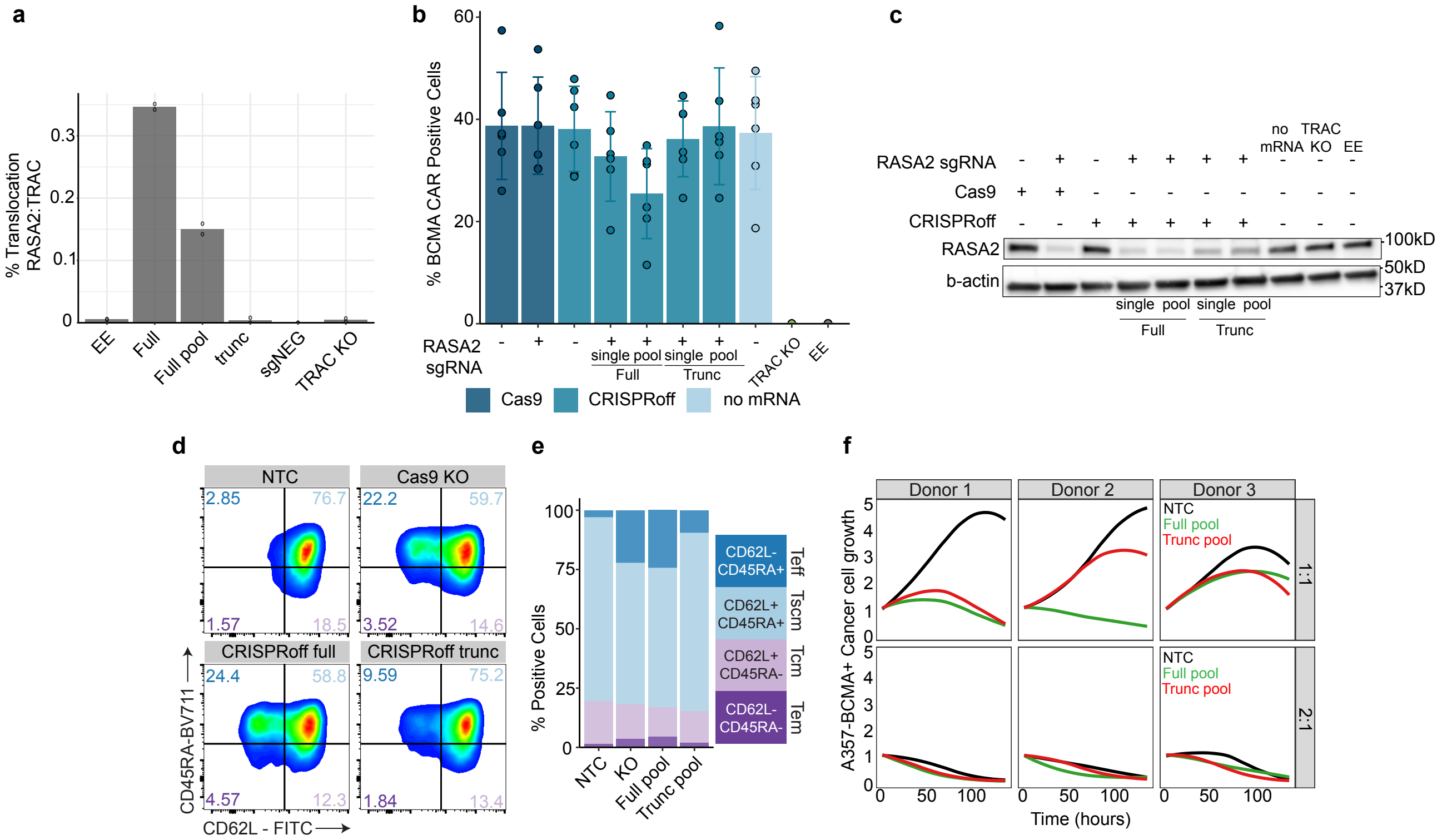

a
% Translocation RASA2:TRAC
EE
Full
Full pool
trunc
sgNEG
TRAC KO
b
% BCMA CAR Positive Cells
RASA2 sgRNA
single pool
single pool
Full
Trunc
TRAC KO
EE
Cas9
CRISPRoff
no mRNA
c
RASA2 sgRNA
Cas9
CRISPRoff
no mRNA
TRAC KO
EE
RASA2
b-actin
100kD
50kD
37kD
single pool
single pool
Full
Trunc
d
NTC
Cas9 KO
CRISPRoff full
CRISPRoff trunc
CD45RA-BV711
CD62L - FITC
e
% Positive Cells
NTC
KO
Full pool
Trunc pool
CD62L- CD45RA+
Teff
CD62L+ CD45RA+
Tscm
CD62L+ CD45RA-
Tcm
CD62L- CD45RA-
Tem
f
Donor 1
Donor 2
Donor 3
NTC
Full pool
Trunc pool
A357-BCMA+ Cancer cell growth
1:1
2:1
Time (hours)

**Supplemental Figure 7: GMP-compatible *S. pyogenes* Cas9 for simultaneous KD and CAR KI. (A)** The graph depicts translocation frequencies between *RASA2* and *TRAC* loci, measured by ddPCR, following transfection of cells with Cas9 RNPs targeting *TRAC* alone or along with CRISPRoff mRNA and the indicated full-length sgRNAs targeting *RASA2*, truncated sgRNAs targeting *RASA2*, or NTC (*n* = 2 donors). **(B)** The graph compares BCMA-specific CAR KI efficiency with Cas9 mRNA or CRISPRoff mRNA used in combination with a NTC or sgRNA targeting *RASA2*. CRISPRoff was electroporated with either one sgRNA or a pool of three sgRNAs targeting the *RASA2* TSS. sgRNAs were either delivered as full-length or truncated (mean ± SD, *n* = 6 donors). **(C)** Western blot images show RASA2 KO with Cas9 mRNA, or CRISPRoff RASA2 KD with either full-length sgRNAs (as one sgRNA or pool of three) or truncated sgRNAs (as one sgRNA or a pool of three). Cells were harvested for protein lysis at 7 days post-electroporation. Data are representative of one donor. **(D,E)** T cell immunophenotypes on day 7 based on CD45RA and CD62L expression, measured by flow cytometry. Data are representative of one donor. **(F)** Graphs show CAR-T cell cytotoxicity as measured by Incucyte analysis after five repetitive stimulations with target cancer cells. Green lines indicate *RASA2*-epi-edited CAR-T cells for cells treated with full-length sgRNAs targeting the *RASA2* TSS, red lines indicated *RASA2*-epi-silenced CAR T cells with truncated sgRNAs targeting the *RASA2* TSS, and black lines are control-edited CAR-T cells. The shaded areas depict the 95% confidence interval for technical replicates (*n* = 3 technical replicates per donor). Each row represents an E:T ratio (top: 1:1, bottom: 2:1).

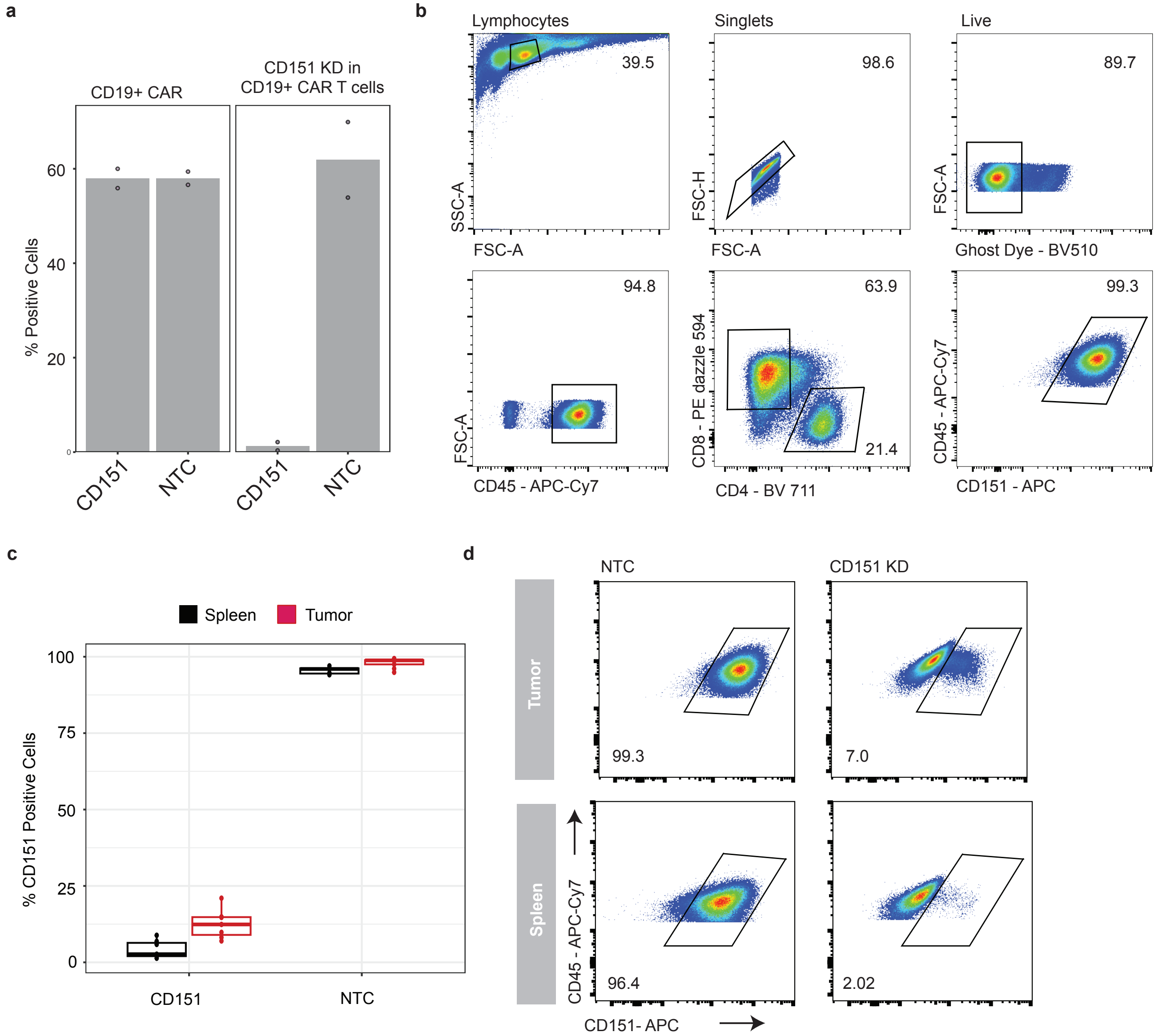

a
CD19+ CAR
CD151 KD in CD19+ CAR T cells
% Positive Cells
0
20
40
60
CD151
NTC
CD151
NTC
b
Lymphocytes
39.5
SSC-A
FSC-A
Singlets
98.6
FSC-H
FSC-A
Live
89.7
FSC-A
Ghost Dye - BV510
94.8
FSC-A
CD45 - APC-Cy7
63.9
21.4
CD8 - PE dazzle 594
CD4 - BV 711
99.3
CD45 - APC-Cy7
CD151 - APC
c
Spleen
Tumor
% CD151 Positive Cells
0
25
50
75
100
CD151
NTC
d
NTC
CD151 KD
Tumor
99.3
7.0
Spleen
96.4
2.02
CD45 - APC-Cy7
CD151- APC

**Supplemental Figure 8: Durability of CRISPRoff silencing in CAR-T cells transferred in vivo. (A)** NSG mice were engrafted with CD19$^+$ A375 melanoma cells via flank injection. Cas12a-compatible CD19-specific *TRAC* CAR-T cells were generated in combination with CRISPRoff mRNA and a pool of three sgRNAs targeting CD151 or a NTC. The left graph shows the CD19+ CAR KI frequency prior to injection and the right graph shows the *CD151* KD prior to injection. CD151-epi-silenced CAR-T cells or control-edited CAR T cells were then transferred into mice via the tail vein 7 days later. **(B)** Representative gating strategy of tumors harvested from mice at 14 days after CAR-T cell injection **(C)**. At 14 days after CAR-T cell injection, the tumor and spleen were harvested from mice, and CD151 expression among CD45$^+$ human T cells was assessed via flow cytometry (tumor: red; spleen: black). **(D)** Representative flow cytometry graphs for T cells from the tumor or spleen that were treated with a NTC or sgRNAs targeting *CD151*.

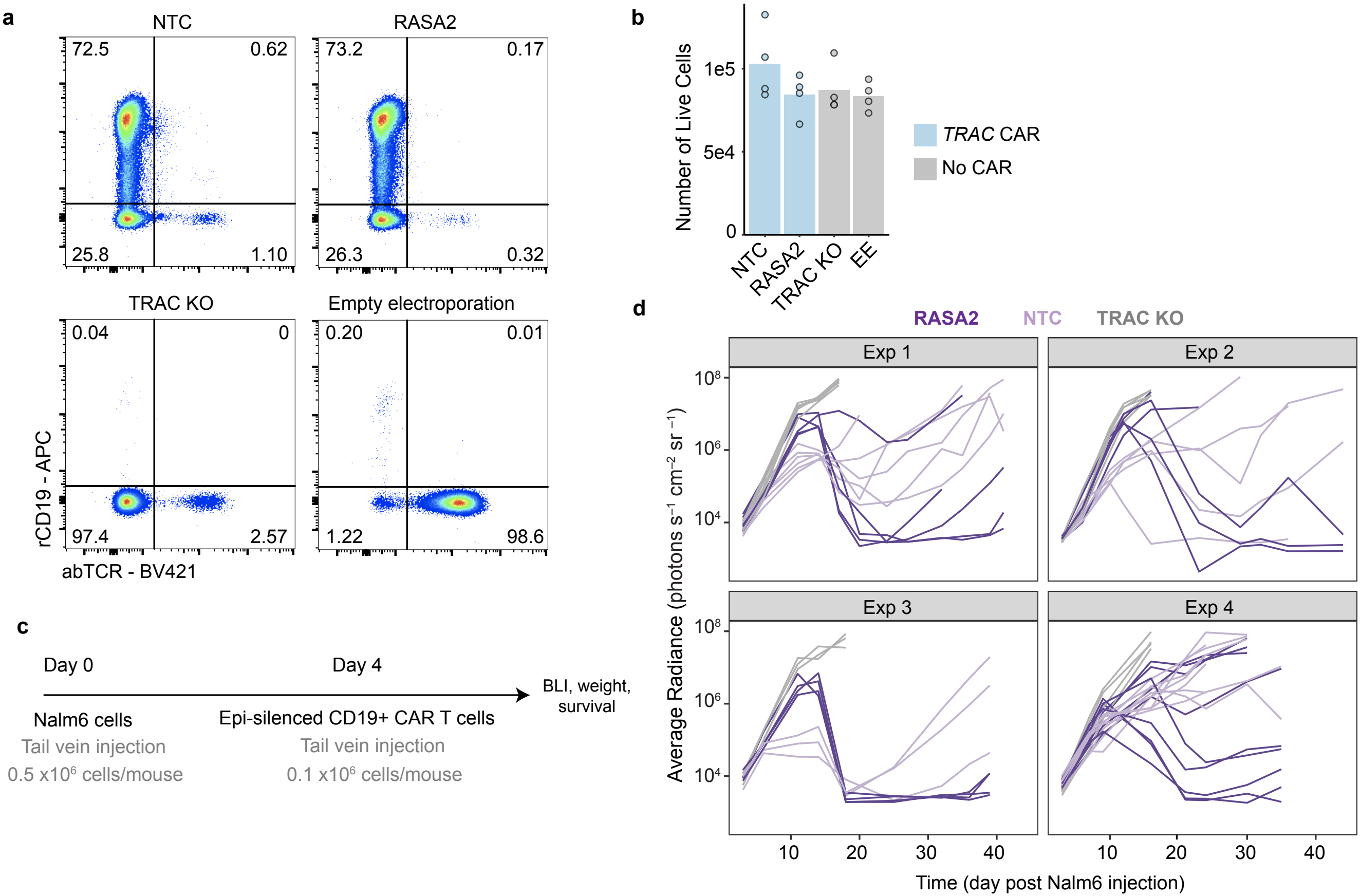
a
NTC
RASA2
72.5
0.62
25.8
1.10
73.2
0.17
26.3
0.32
TRAC KO
Empty electroporation
0.04
0
97.4
2.57
0.20
0.01
1.22
98.6
rCD19 - APC
abTCR - BV421
b
Number of Live Cells
1e5
5e4
0
NTC
RASA2
TRAC KO
EE
TRAC CAR
No CAR
c
Day 0
Day 4
Nalm6 cells
Tail vein injection
0.5 x10^6 cells/mouse
Epi-silenced CD19+ CAR T cells
Tail vein injection
0.1 x10^6 cells/mouse
BLI, weight, survival
d
RASA2
NTC
TRAC KO
Exp 1
Exp 2
Exp 3
Exp 4
Average Radiance (photons s^-1 cm^-2 sr^-1)
10^8
10^6
10^4
10
20
30
40
Time (day post Nalm6 injection)

**Supplemental Figure 9: RASA2 epi-silenced CAR T cells improve tumor control in vivo. (A)** Flow cytometry data for levels of CD19-specific CAR positive and TCRα/β KO T cells immediately prior to injection. An equal number of $CAR^+$ T cells was injected into each mouse. Data are representative of one donor. **(B)** Live cell counts collected nine days after electroporation between cells that have a *TRAC* CAR KI (blue) or no CAR KI (grey). EE: empty electroporation. ($n$ = 4 donors). **(C)** Experimental timeline. **(D)** Bioluminescence images data for tumor growth over time in NSG mice engrafted with $0.5 \times 10^6$ Nalm6 cells and treated with $0.1 \times 10^6$ $CAR^+$ T cells that underwent treatment for *RASA2*-epi-silencing.

# Supplemental Figure 7C

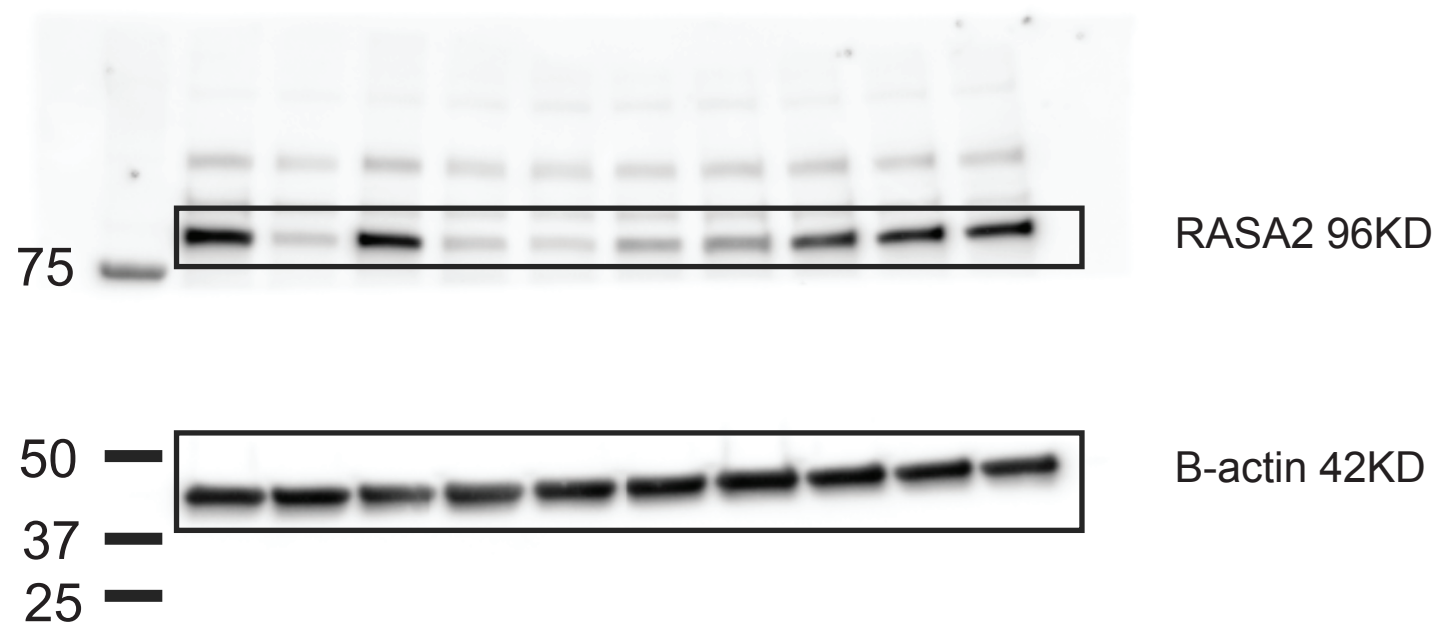

Supplement: Supplementary file 1 [file 41587_2025_2856_MOESM1_ESM.pdf]
