# Supplementary material for: Integrated epigenetic and genetic programming of primary human T cells
Source: Nat Biotechnol. 2025 Oct 21;44(9):1470–81. doi: 10.1038/s41587-025-02856-w (PMC13336058; doi:10.1038/s41587-025-02856-w)
Supplement: Supplementary file 2 [file 41587_2025_2856_MOESM2_ESM.pdf]

nature portfolio

Corresponding author(s): Luke A. Gilbert, Brian R. Shy, Alex Marson, Justin Eyquem

Last updated by author(s): 9/10/2025

Reporting Summary

Nature Portfolio wishes to improve the reproducibility of the work that we publish. This form provides structure for consistency and transparency in reporting. For further information on Nature Portfolio policies, see our Editorial Policies and the Editorial Policy Checklist.

Statistics

For all statistical analyses, confirm that the following items are present in the figure legend, table legend, main text, or Methods section.

n/a	Confirmed	
☐	☒	The exact sample size (*n*) for each experimental group/condition, given as a discrete number and unit of measurement
☐	☒	A statement on whether measurements were taken from distinct samples or whether the same sample was measured repeatedly
☐	☒	The statistical test(s) used AND whether they are one- or two-sided<br>*Only common tests should be described solely by name; describe more complex techniques in the Methods section.*
☐	☒	A description of all covariates tested
☐	☒	A description of any assumptions or corrections, such as tests of normality and adjustment for multiple comparisons
☐	☒	A full description of the statistical parameters including central tendency (e.g. means) or other basic estimates (e.g. regression coefficient) AND variation (e.g. standard deviation) or associated estimates of uncertainty (e.g. confidence intervals)
☐	☒	For null hypothesis testing, the test statistic (e.g. *F*, *t*, *r*) with confidence intervals, effect sizes, degrees of freedom and *P* value noted<br>*Give P values as exact values whenever suitable.*
☒	☐	For Bayesian analysis, information on the choice of priors and Markov chain Monte Carlo settings
☒	☐	For hierarchical and complex designs, identification of the appropriate level for tests and full reporting of outcomes
☒	☐	Estimates of effect sizes (e.g. Cohen's *d*, Pearson's *r*), indicating how they were calculated

Our web collection on statistics for biologists contains articles on many of the points above.

Software and code

Policy information about availability of computer code

Data collection	Attune NTX Cytometer control software was used to collect flow cytometry data (v6.0.1). Incucyte Zoom software was used to count RFP positive cells in the cancer cell killing assays (v.2021B). Western blots were imaged on the Azure Biosystems 600 imaging system. RT-qPCR was performed on the Quant studio Real-Time PCR System. Mouse imaging was collected on an IVIS Lumina S5 (Revvity).
Data analysis	Flow cytometry data was gated with FlowJo v 10.9.0. For multiplex experiments, FlowJo workspaces were read into R (v.4.3.0) using packages 'flowCore', 'CytoML', and 'openCyto'. RNA-seq reads were aligned to the hg38 reference genome using STAR (version 2.7.3a); RNA counts were measured with the nf-core pipeline (v 3.18). Raw WGBS-seq FASTQ files were processed using the nf-methylseq:2.6.0 pipeline, differential CpG DNA methylation analysis was performed using the methylKit R package. Differentially methylated regions were visualized as Manhattan plots generated in R or displayed in IGV to visualize base level methylation. Plots were generated by ggplot v3.4.4 unless otherwise specified. R code used in this manuscript for RNA-seq analysis and WGBS analysis are available on the Gilbert lab GitHub page: https://github.com/GilbertLabUCSF/T_Cell_CRISPRoff. Bioluminescence from live mouse imaging for in vivo experiments was analyzed with the Living Image software (v.4.7.3). Figures were compiled with Abode Illustrator v27.5.

For manuscripts utilizing custom algorithms or software that are central to the research but not yet described in published literature, software must be made available to editors and reviewers. We strongly encourage code deposition in a community repository (e.g. GitHub). See the Nature Portfolio guidelines for submitting code & software for further information.

Data

Policy information about availability of data

All manuscripts must include a data availability statement. This statement should provide the following information, where applicable:

- Accession codes, unique identifiers, or web links for publicly available datasets
- A description of any restrictions on data availability
- For clinical datasets or third party data, please ensure that the statement adheres to our policy

The data discussed in the publication have been deposited on NCBI's Gene Expression Omnibus (Goudy et al., 2025) and are accessible through GEO Series accession number GSE306915 (RNA-sequencing) and GSE306917 (WGBS).

Research involving human participants, their data, or biological material

Policy information about studies with human participants or human data. See also policy information about sex, gender (identity/presentation), and sexual orientation and race, ethnicity and racism.

Reporting on sex and gender	Peripheral blood monoculear cells (PMBCs) were purchased from StemCell Technologies from healthy male and female donors. No preference was given to sex or age for this study. Cells were purchased on a weekly basis so experiments throughout this study used different donors.
Reporting on race, ethnicity, or other socially relevant groupings	No preference was given to race or ethnicity from donors. Age was not a factor in our selection of donors. We chose donors based on StemCell's criteria for eligibility which is a minimum of 18 years old and a maximum of 55 years old.
Population characteristics	*Describe the covariate-relevant population characteristics of the human research participants (e.g. age, genotypic information, past and current diagnosis and treatment categories). If you filled out the behavioural & social sciences study design questions and have nothing to add here, write "See above."*
Recruitment	PBMCs were purchased from StemCell Technologies who were wholly responsible for the recruitment of donors.
Ethics oversight	StemCell Technologies collects PBMCs from healthy donors under approved protocols by the StemCell Technologies IRB. STEMCELL provides the following information, "Subjects are voluntary, healthy donors, recruited from the general community, and compensated for their time and effort. Criteria for entry into the program includes age (minimum: 18 years old; maximum: 55 years old or higher, depending on the donor site), and donors withoutany pre-existing conditions such as cancer, cardiac, lung, blood, or autoimmune disorders. Subjects are recruited without regard for ethnicity, although US citizenship is required."

Note that full information on the approval of the study protocol must also be provided in the manuscript.

Field-specific reporting

Please select the one below that is the best fit for your research. If you are not sure, read the appropriate sections before making your selection.

☒ Life sciences ☐ Behavioural & social sciences ☐ Ecological, evolutionary & environmental sciences

For a reference copy of the document with all sections, see nature.com/documents/nr-reporting-summary-flat.pdf

Life sciences study design

All studies must disclose on these points even when the disclosure is negative.

Sample size	For in vitro studies, all experiments were done with at least two independent, healthy donors. For in vivo studies, no statistical method was used to predetermine sample size. We had at least n=5 mice per group, based on previously published data (PMID: 36002574, PMID: 36008610).
Data exclusions	Results show all data points collected from the experiments described in the manuscript.
Replication	Human biological replicates (independent, healthy donors) were used in all experiments to ensure reproducibility. Replicate experiments were performed as described in figure legends and methods.
Randomization	Mice were randomized based on tumor size or BLI signal before T cell injection to ensure equal tumor burden distribution across groups. For in vitro studies, controls and treatments were performed in matched cells from the same donor (i.e. control targeting sgRNA and gene of interest sgRNAs).
Blinding	The investigators were not blinded to allocation during experiments and outcome assessment as the individual who designed the experiment was often involved in performing the experiment. All experiments were conducted with methodologies to reduce biases such as use of mutichannel pipetting to ensure equal treatment of all samples. Data collection was not blinded but was measured with objective methodologies such as flow cytometry, incucyte, RNA-seq, RT-qPCR.

Reporting for specific materials, systems and methods

We require information from authors about some types of materials, experimental systems and methods used in many studies. Here, indicate whether each material, system or method listed is relevant to your study. If you are not sure if a list item applies to your research, read the appropriate section before selecting a response.

Materials & experimental systems

n/a	Involved in the study
☐	☒ Antibodies
☐	☒ Eukaryotic cell lines
☒	☐ Palaeontology and archaeology
☐	☒ Animals and other organisms
☒	☐ Clinical data
☒	☐ Dual use research of concern
☒	☐ Plants

Methods

n/a	Involved in the study
☒	☐ ChIP-seq
☐	☒ Flow cytometry
☒	☐ MRI-based neuroimaging

Antibodies

Antibodies used

For Flow Cytometry:
PE anti-human CD81 (Clone 5A6, Biolegend, Cat# 349506), FITC anti-human CD55 (Clone JS11, Biolegend, Cat# 311306), APC anti-human CD151 (Clone 50-6, Biolegend, Cat#350406), Brilliant Violet 605 anti-human CD44 (Clone IM7, Biolegend, Cat# 103049), PE/Dazzle 594 anti-human CD46 (Clone TRA-2-10, Biolegend, Cat# 352412), APC anti-human PD1 (Clone EH12.2H7, Biolegend, Cat# 329908), PE anti-human CD45 (Clone H130, Biolegend, Cat# 304008). GhostDye 780 (TonboBiosciences Cat# 13-0865-T500), APC Streptavidin (Biolegend, Cat# 405207). BV711 anti-human CD4 (Clone RPA-T4, Biolegend, Cat #300558). Pacific Blue anti-human CD8 (Clone SK1, Biolegend, Cat #344718). BV711 anti-human CD45RA (Clone HI100, Biolegend, Cat #304138). FITC anti-human CD62L (Clone DREG-56, Biolegend, Cat #304838). PE/Cy7 anti-human CD5 (Clone UCHT2, Biolegend, Cat #300622). BV711 anti-human LAG3 (Clone 11C3C65, Biolegend, Cat #369320). APC/Cy7 anti-human CD39 (Clone A1, Biolegend, Cat# 328226). Af488 anti-human FOXP3 (Clone 206D, Biolegend, Cat#320112).

For Western Blotting:
RASA2 (Sigma Aldrich, HPA035375), B-actin rabbit monoclonal (horseradish peroxidase (HRP) conjugate) (Cell Signaling 5125), goat-anti-rabbit IgG-HRP (Jackson ImmunoResearch 111-036-045), and goat-anti-mouse IgG-HRP (sc-2005 Santa Cruz Biotechnology).

From Acrobiosystems:
Biotinylated Human BCMA / TNFRSF17 Protein (Cat# BCA-H82E4-200ug), Biotinylated Human CD19 (20-291) Protein (Cat# CD9-H82F6-25ug).

Validation

All flow cytometry antibodies were validated and quality tested on the manufacturer website including a histogram of positive and negative cells stained with the respective product. Prior to using each antibody, we performed testing on the cell types of interest, using relevant fixation methods if applicable, to determine specificity and robust detection without significant spillover into the channels of other markers used in the study. Unless otherwise stated, all flow antibodies were used at a 1:50X dilution.

-PE anti-human CD81 (Clone 5A6, Biolegend, Cat# 349506) was validated here: https://www.biolegend.com/en-us/products/pe-anti-human-cd81-tapa-1-antibody-6767
- FITC anti-human CD55 (Clone JS11, Biolegend, Cat# 311306) was validated here: https://www.biolegend.com/en-us/products/fitc-anti-human-cd55-antibody-1794
- APC anti-human CD151 (Clone 50-6, Biolegend, Cat#350406) was validated here: https://www.biolegend.com/en-us/products/apc-anti-human-cd151-peta-3-antibody-6962
- Brilliant Violet 605 anti-human CD44 (Clone IM7, Biolegend, Cat# 103049) was validated here: https://www.biolegend.com/en-us/products/brilliant-violet-650-anti-mouse-human-cd44-antibody-8923
- PE/Dazzle 594 anti-human CD46 (Clone TRA-2-10, Biolegend, Cat# 352412) was validated previously at Biolegend but has since been discontinued.
- APC anti-human PD1 (Clone EH12.2H7, Biolegend, Cat# 329908) was validated here: https://www.biolegend.com/en-us/products/apc-anti-human-cd279-pd-1-antibody-4413
- PE anti-human CD45 (Clone H130, Biolegend, Cat# 304008) was validated here: https://www.biolegend.com/en-us/products/pe-anti-human-cd45-antibody-708
- APC Streptavidin (Biolegend, Cat# 405207) was validated here: https://www.biolegend.com/en-us/products/apc-streptavidin-1470
- BV711 anti-human CD4 (Clone RPA-T4, Biolegend, Cat #300558) was validated here: https://www.biolegend.com/en-us/products/brilliant-violet-711-anti-human-cd4-antibody-10435
- Pacific Blue anti-human CD8 (Clone SK1, Biolegend, Cat #344718) was validated here: https://www.biolegend.com/en-us/products/pacific-blue-anti-human-cd8-antibody-6509
- BV711 anti-human CD45RA (Clone HI100, Biolegend, Cat #304138) was validated here: https://www.biolegend.com/en-us/products/brilliant-violet-711-anti-human-cd45ra-antibody-7937
- FITC anti-human CD62L (Clone DREG-56, Biolegend, Cat #304838) was validated here: https://www.biolegend.com/en-us/products/fitc-anti-human-cd62l-antibody-651
- PE/Cy7 anti-human CD5 (Clone UCHT2, Biolegend, Cat #300622) was validated here: https://www.biolegend.com/en-us/products/pe-cyanine7-anti-human-cd5-antibody-4626
- BV711 anti-human LAG3 (Clone 11C3C65, Biolegend, Cat #369320) was validated here: https://www.biolegend.com/en-us/

products/brilliant-violet-711-anti-human-cd223-lag-3-antibody-14878
- APC/Cy7 anti-human CD39 (Clone A1, Biolegend, Cat# 328226) was validated here: https://www.biolegend.com/en-us/products/apc-cyanine7-anti-human-cd39-antibody-12925
- Af488 anti-human FOXP3 (Clone 206D, Biolegend, Cat#320112) was validated here: https://www.biolegend.com/en-us/products/alexa-fluor-488-anti-human-foxp3-antibody-2914

Western Blot antibodies:
-RASA2 (Sigma Aldrich, HPA035375) was used at a 1:4,000X dilution: https://www.sigmaaldrich.com/US/en/product/sigma/hpa035375?srsltid=AfmBOop1ICSiauf2rlNb0QCKZy_kSnn_lE3UqdZYHMuD9U54yu6-LsFX
- B-actin rabbit monoclonal (horseradish peroxidase (HRP) conjugate) (Cell Signaling 5125) was used at a 1:20,000X dilution: https://www.cellsignal.com/products/antibody-conjugates/b-actin-13e5-rabbit-mab-hrp-conjugate/5125?srsltid=AfmBOooVEHkm5d0xOZVEKEyz7-ozWoqVyzrl0bdch08Im_HC4alFVa9E
- goat-anti-rabbit IgG-HRP (Jackson ImmunoResearch 111-036-045): https://www.jacksonimmuno.com/catalog/products/111-036-045
-goat-anti-mouse IgG-HRP (sc-2005 Santa Cruz Biotechnology): https://www.scbt.com/p/goat-anti-mouse-igg-hrp?srsltid=AfmBOopCJyMCnWUmdWEg54HMJmyG_uTy6MOqn_cYWZfzWkYhigaWOI1C
- Biotinylated Human BCMA / TNFRSF17 Protein (Cat# BCA-H82E4-200ug): https://www.acrobiosystems.com/P2481-Biotinylated-Human-BCMA--TNFRSF17-Protein-HisAvitag%E2%84%A2-premium-grade.html
- Biotinylated Human CD19 (20-291) Protein (Cat# CD9-H82F6-25ug): https://www.acrobiosystems.com/P5428-Biotinylated-Human-CD19-%2820-291%29-Protein-FcAvitag%E2%84%A2-premium-grade.html

Eukaryotic cell lines

Policy information about cell lines and Sex and Gender in Research

Cell line source(s)

A375 (ATCC, CRL-1619)
A375-CD19 (generated in PMID: 36002574)
A375-BCMA - a gift from Justin Eyquem
Nalm6 expressing luciferase and GFP, varying levels of CD19 - Gift from Justin Eyquem and published in PMID: 36002574. Originally purchased from ATCC (CRL-3273)

Authentication

All cells were originally purchased from ATCC but no further validation was performed. Relevant antigen expression was routinely measured via flow cytometry.

Mycoplasma contamination

All cell lines used were tested for mycoplasma and had a negative result.

Commonly misidentified lines (See ICLAC register)

Name any commonly misidentified cell lines used in the study and provide a rationale for their use.

Animals and other research organisms

Policy information about studies involving animals; ARRIVE guidelines recommended for reporting animal research, and Sex and Gender in Research

Laboratory animals

8-10 week old female NOD-SCID-IL2rg-/- (NSG) mice were purchased from JAX Lab (Mus musculus, Strain #005557).

Wild animals

This study did not involve wild animals.

Reporting on sex

All mice used for these experiments were female.

Field-collected samples

No field-collected samples were used in this study.

Ethics oversight

Mice were used in accordance with ethical guidelines approved by the UCSF Institutional Animal Car and Use Committee. Specifically, the IACUC protocol used in the Marson lab is #195573.

Note that full information on the approval of the study protocol must also be provided in the manuscript.

Plants

Seed stocks

Report on the source of all seed stocks or other plant material used. If applicable, state the seed stock centre and catalogue number. If plant specimens were collected from the field, describe the collection location, date and sampling procedures.

Novel plant genotypes

Describe the methods by which all novel plant genotypes were produced. This includes those generated by transgenic approaches, gene editing, chemical/radiation-based mutagenesis and hybridization. For transgenic lines, describe the transformation method, the number of independent lines analyzed and the generation upon which experiments were performed. For gene-edited lines, describe the editor used, the endogenous sequence targeted for editing, the targeting guide RNA sequence (if applicable) and how the editor was applied.

Authentication

Describe any authentication procedures for each seed stock used or novel genotype generated. Describe any experiments used to assess the effect of a mutation and, where applicable, how potential secondary effects (e.g. second site T-DNA insertions, mosiacism, off-target gene editing) were examined.

Flow Cytometry

Plots

Confirm that:

☒ The axis labels state the marker and fluorochrome used (e.g. CD4-FITC).

☐ The axis scales are clearly visible. Include numbers along axes only for bottom left plot of group (a 'group' is an analysis of identical markers).

☒ All plots are contour plots with outliers or pseudocolor plots.

☒ A numerical value for number of cells or percentage (with statistics) is provided.

Methodology

Sample preparation	For flow cytometry, between 0.1-0.5 million cells were collected from culture and washed with FACS staining buffer (PBS + EDTA + FCS). Cells were stained with a 1:50 dilution containing the relevant antibodies for 20 minutes in the dark at 4C. Samples were then washed twice with FACS buffer before running on the Attune NXT Cytometer. For FOXP3 staining, The Biolegend FoxP3 Fix/Perm kit (Biolegend, #421403) was used for staining according to the manufacturer protocol. Cells were washed in EasySep buffer prior to extracellular staining, and stained with relevant extracellular antibodies for 20 minutes at 4C in the dark. After fixing and permeabilizing according to the kit, intracellular staining was performed with Af488 anti-human FOXP3 Antibody (Biolegend, #320112) diluted 1:50 in permeabilization buffer for 30 minutes at room temperature. Cells were subsequently washed in permeabilization buffer and resuspended in EasySep buffer before running on the ThermoFisher Attune NxT flow cytometer.
Instrument	All flow cytometry was conducted on an Attune NXT Cytometer with a 96-well autosampler (Thermo Fisher Scientific).
Software	Flow cytometry analysis was performed using FlowJo v10.9.0 software. Data was exported to R and plots were made with ggplot2 (v3.4.4). For multiplex experiments, FlowJo workspaces were read into R (v.4.3.0) using packages 'flowCore', 'CytoML', and 'openCyto', and graphs were made with ggplot2 (v3.4.4). Figures were compiled in Adobe Illustrator v27.5.
Cell population abundance	*Describe the abundance of the relevant cell populations within post-sort fractions, providing details on the purity of the samples and how it was determined.*
Gating strategy	For all experiments, viable lymphocytes were gated by FSC-A/SSC-A. Singlets were then gated by FSC-A/FSC-H. A viability dye was then used to gate live cells. Positive populations were determined by the unstained samples. In co-culture experiments and in vivo experiments, T cells were defined as CD45+ and RFP negative.

☒ Tick this box to confirm that a figure exemplifying the gating strategy is provided in the Supplementary Information.
