# Supplementary material for: Integrated epigenetic and genetic programming of primary human T cells
Source: Nat Biotechnol. 2025 Oct 21;44(9):1470–81. doi: 10.1038/s41587-025-02856-w (PMC13336058; doi:10.1038/s41587-025-02856-w)

Main Fig 5C

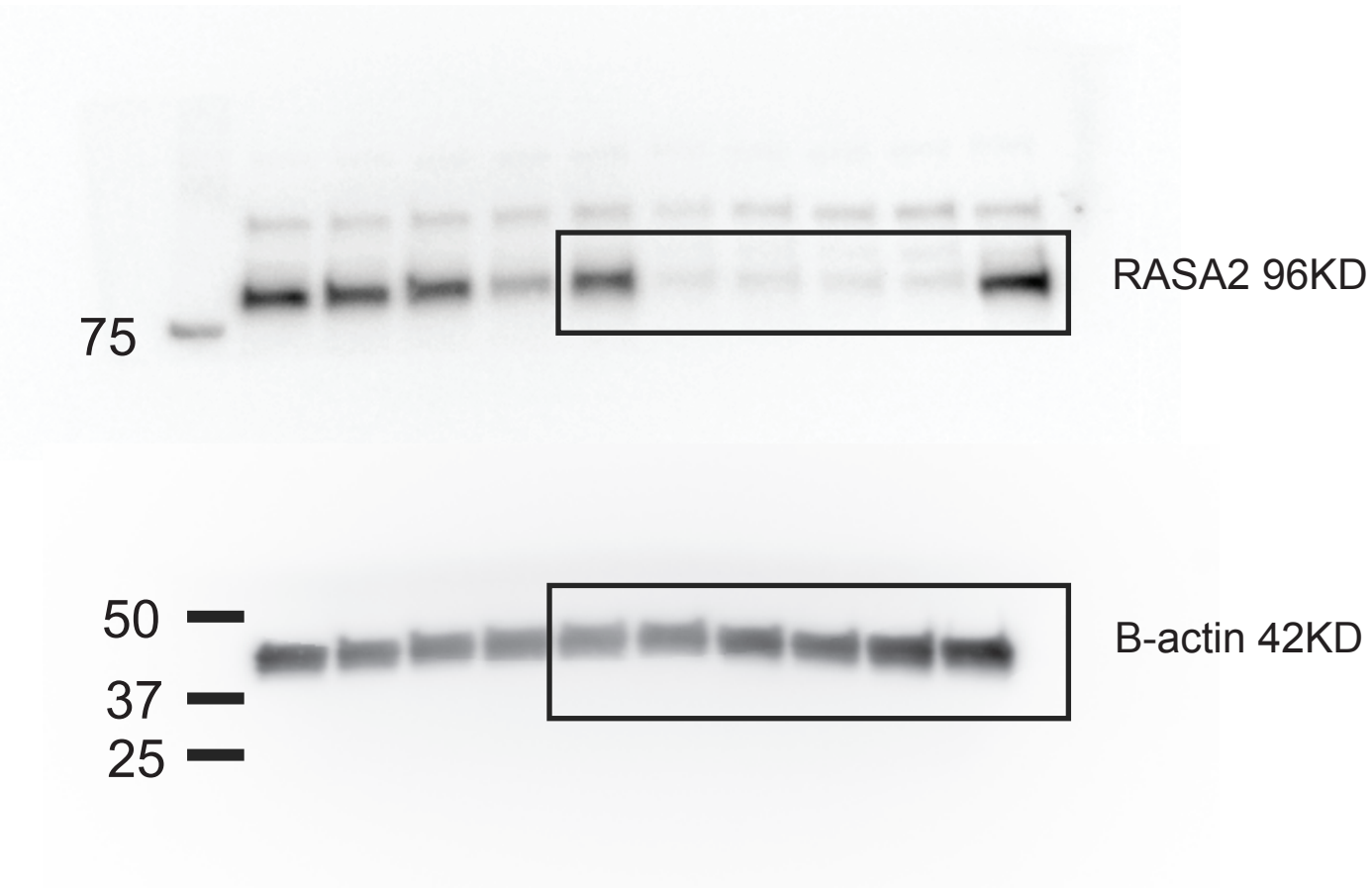

Main Fig 5I

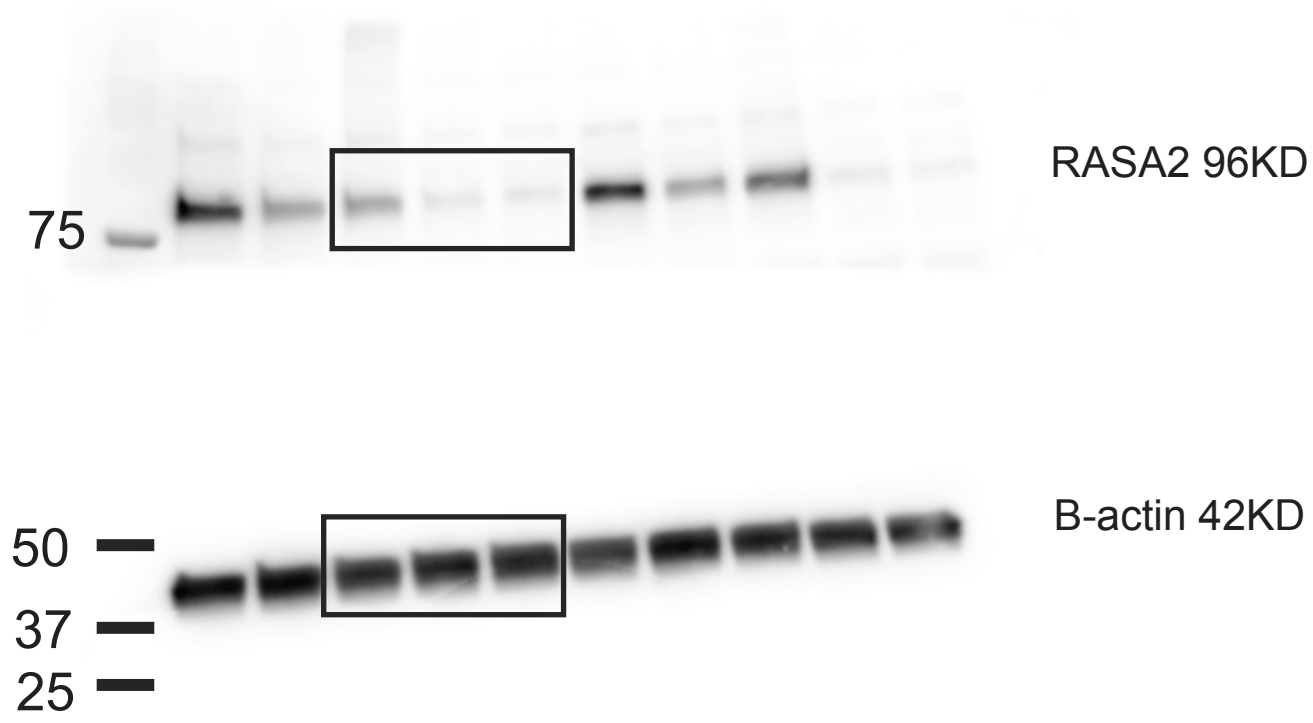

Supplemental Figure 7C

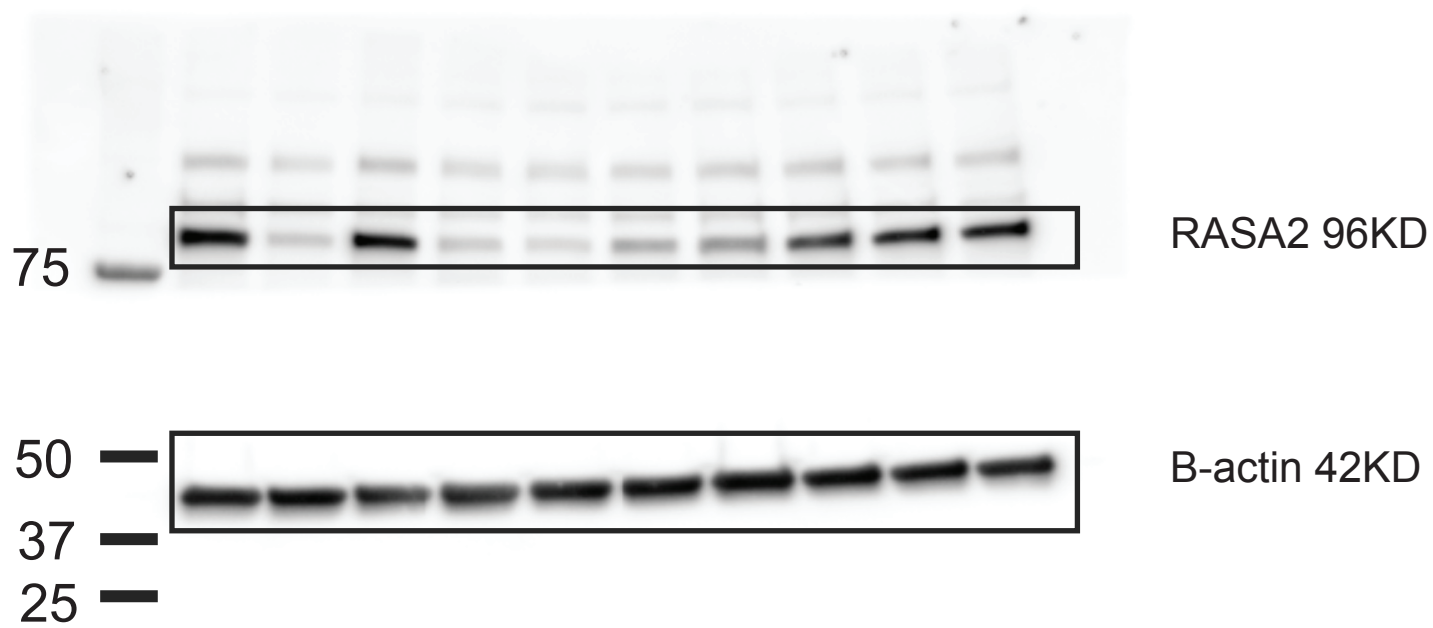

Supplement: Supplementary file 3 [file 41587_2025_2856_MOESM4_ESM.pdf]
